# Nocebo effects of a simplified package leaflet compared to unstandardised oral information and a standard package leaflet: a pilot randomised controlled trial

**DOI:** 10.1186/s13063-019-3565-3

**Published:** 2019-07-26

**Authors:** Barbara Prediger, Esther Meyer, Roland Büchter, Tim Mathes

**Affiliations:** Institute for Research in Operative Medicine, Witten/Herdecke University, Ostmerheimer Str. 200, 51109 Cologne, Germany

**Keywords:** Randomised controlled trial, Nocebo effect, Patient information leaflets

## Abstract

**Background:**

The term “nocebo effect” describes the phenomenon that the mere knowledge and anticipation of possible negative consequences of an intervention can increase the probability of experiencing these consequences. Our objective was to assess whether different information presentations on adverse events (AEs) in package information leaflets (PILs) could influence the nocebo effect.

**Methods:**

We included patients undergoing orthopaedic surgery in this pilot randomised controlled trial (pRCT). Patients were assigned by random, computerised and centralised allocation to one of three groups: Simplified-PIL, No-PIL or Standard-PIL on ibuprofen. The Simplified-PIL was written in plain language, and AEs were reported with a focus on avoiding biased risk perception. Only the outcome assessment was blinded.

**Results:**

We included 35, 33 and 34 patients in the Simplified-PIL, No-PIL and Standard-PIL groups, respectively. All patients were included in the intention-to-treat analysis. Six patients in the Simplified-PIL, four in the No-PIL and eight in the Standard-PIL group reported an AE. This corresponds to relative risks of 0.80 (95% confidence interval (CI) 0.27–1.90) for the Simplified-PIL and 0.50 (95% CI 0.14–1.46) for the No-PIL compared with the Standard-PIL group. The Simplified-PIL increased knowledge, reduced anxiety and improved adherence, although statistical uncertainty was high for all of these outcomes.

**Conclusions:**

This pRCT provides the first hints on the way information on AEs is reported in PILs can affect the nocebo effect. This pRCT shows that a definitive RCT is feasible. If the results are confirmed in a definitive large RCT, a revision of the current practice for designing PILs should be considered.

**Trial registration:**

ClinicalTrials.gov identifier: NCT03428035. Registered 2 February 2018.

**Electronic supplementary material:**

The online version of this article (10.1186/s13063-019-3565-3) contains supplementary material, which is available to authorized users.

## Background

The term “nocebo effect” describes the phenomenon that the mere knowledge and anticipation of possible negative consequences of an intervention can increase the probability of experiencing these consequences [1]. Systematic reviews of randomised controlled trials (RCTs) have shown that adverse events (AEs) often occur in placebo groups and active treatment groups to a similar extent [2–4].

The nocebo effect can be induced in the context of either an inert treatment (e.g., placebo) or an active treatment (e.g., drug therapy). In the clinical context, the nocebo effect (undesired AEs) of active treatments is particularly important [5]. Studies suggest that the nocebo effect has a neural basis and operates in a psychosomatic way [6]. Recent research indicates that the nocebo effect can be influenced by the way information on side effects of a treatment is provided [7, 8]. Via cognitive processes like expectation or conditioning, negative information on side effects may act as a moderator on the occurrence of AEs [9].

The “additional” side effects can have a negative impact on patients’ quality of life and on the effectiveness of treatments [7]. Moreover, expecting or experiencing adverse reactions can lead to non-adherence and discontinuation of the therapy and to costs for additional treatment to reduce the symptoms [10]. The potential of unintentionally causing a nocebo effect by giving information can be an ethical and legal dilemma. On one hand, not informing patients of possible AEs could protect them from developing symptoms that could be avoided. On the other hand, it is not acceptable and often conflicts with legal requirements not to deprive patients of their right to information and involvement in their treatment.

The most common way to provide written information about medication and its AEs is by a package information leaflet (PIL) [11–13]. Currently, the standard PILs in Europe contain a broad spectrum of possible side effects that are presented in a way that might lead to an inaccurate and increased risk perception [14]. The resulting (stronger) anticipation of AEs may induce a nocebo effect, namely an actual rise of AEs experienced by patients [15].

The primary objective of this pilot randomised controlled trial (pRCT) was to assess whether the type and style of presentation of AE information in PILs could influence the nocebo effect (frequency of AEs). For this purpose, we compared a newly designed PIL that was designed with a focus on comprehensibility and neutral risk perception with unstandardised oral information and a European Union (EU) directive [16]-compliant standard PIL. A second aim was to analyse whether the different types of AE information have an impact on patient adherence.

## Materials and methods

### Study design

This study is a three-arm (1:1:1 allocation ratio) parallel randomised controlled pilot trial. The study is registered in ClinicalTrials.gov (NCT03428035) and the German Clinical Trials Register (DRKS00013923). The full study protocol (German only) and explanation for deviations from protocol can be found in Additional files 1 and 2. The ethics committee of the University of Witten/Herdecke approved the study. The study was performed in accordance with the Declaration of Helsinki and the International Council for Harmonisation standards for Good Clinical Practice [17, 18]. The results of the RCT are reported in accordance with the Consolidated Standards of Reporting Trials (CONSORT) extension for randomised pilot and feasibility trials [19].

### Participants and setting

The study was performed at a tertiary hospital in Cologne, Germany, in the department of trauma surgery and orthopaedics (Cologne-Merheim Hospital, the teaching hospital of the University of Witten/Herdecke). We recruited patients between April and September 2018. Patients had to satisfy the following inclusion criteria:

- at least 18 years old
- scheduled for elective orthopaedic surgery
- scheduled to receive only ibuprofen 600 mg for pain relief after discharge
- able to understand German
- cognitive ability to give consent and answer questions.

Because we assumed that these patients are conditioned by previous experiences with pain medication and therefore the change of expectations caused by the Simplified-PIL would be low, we excluded patients who took pain medication regularly for chronic pain. We also excluded patients who took medication that can cause AEs similar to those of ibuprofen and who had an illness that could cause symptoms similar to the AEs of ibuprofen (e.g., gastrointestinal or neurological diseases) because the sample size was low and thus randomisation would not have ensured balance of these variables, which had a high potential for confounding. In addition, we excluded patients who had multiple fractures or went to inpatient rehabilitation after surgery since it would not have been possible to interview these patients three days after surgery. We identified eligible patients at the pre-operation visit (outpatient surgery) or by screening the patient lists of hospital units (inpatients). All patients gave their written informed consent to take part in the study before inclusion.

### Interventions

We randomly assigned patients to one of three groups:

- Simplified-PIL
- Standard-PIL
- No-PIL.

Patients allocated to the “Simplified-PIL” group received a newly designed PIL for ibuprofen 600 mg. The design of the PIL was informed by knowledge from risk communication research, research on patient preferences about PILs and evidence-based health information guidelines (e.g., [20–25]). The main design criteria were comprehensibility and descriptions that avoided incorrect risk perception on AEs.

In order to find valid frequency data, we performed a focused literature search on Cochrane systematic reviews of RCTs of ibuprofen versus placebo (e.g., [26]). As there can be a difference in the type (e.g., gastrointestinal bleeding) of AEs and frequency of AEs depending on intake period, we extracted the number of AEs only from studies of ibuprofen use for post-surgical pain relief (i.e., short period).

We used plain language for all descriptions in the Simplified-PIL and avoided redundant and self-explaining information (e.g., consulting a physician in case of serious situations). For non-serious AEs, we reported frequencies only for those with sufficient underlying certainty of evidence. We reported AE frequencies per 100 patients. We report the difference in the number of AEs between patients receiving ibuprofen and patients receiving placebo because these AEs can be considered to be the share of AEs actually causally related to ibuprofen [27]. In contrast, the standard package leaflets provide the total frequency of AEs in patients who received ibuprofen and thus counts general complaints as AEs from ibuprofen (e.g., headaches and diarrhoea). We report how many people did not experience an AE (positive framing) in addition to how many people experience an AE [28]. For illustration, we used pictograms [29]. For non-serious AEs with uncertain evidence, we stated that the evidence for these AEs is insufficient.

We reported all known serious AEs irrespective of the certainty of the evidence. Serious AEs are typically rare and RCTs usually are not powered for valid estimations of their frequency. As expected, we could identify only very low-certainty evidence for frequency data on serious AEs. Because of this uncertainty, we reported serious AEs without providing numeric information on frequencies. Instead, we mentioned only their possible occurrence and expressed the uncertainty of the evidence.

In order to avoid biased information on the benefit-harm ratio, we included a short description on the effectiveness of ibuprofen for pain relief after surgery [25, 30].

The Simplified-PIL was pilot-tested and modified according to the feedback from six patients. A translated version of the Simplified-PIL can be found in Additional file 3.

Participants allocated to the “Standard-PIL” group received a copy of a standard EU directive–compliant PIL for ibuprofen 600 mg [16, 31]. The Standard-PIL includes information on the drug (e.g., ingredients), information necessary before taking the drug (e.g., interactions with other medicines and pregnancy) and information on dosage, possible adverse effects and additional information (e.g., storage). In the Standard-PIL, all possible AEs are listed and frequencies are described by using fixed terminology for certain frequency ranges (e.g., common: at least 1/10 to 1/100). There is no standardisation on format, structure, layout or comprehensibility. The Standard-PIL used can be found in Additional file 4.

Table 1 contains examples for the frequency information on AEs as provided in the Simplified-PIL and the Standard-PIL. The PILs were provided before surgery and the patients were asked to carefully read them. The No-PIL group received unstandardised oral information about their medication as routinely provided in the hospital. This usually includes information on intake but little or no information on AEs.

**Table 1:** Depiction of adverse events in both package information leaflets

Simplified-PIL	Standard-PIL	
*Adverse events*	*Adverse events*	
Gastrointestinal conditions Ibuprofen caused stomach and bowel discomforts in about 1 in 100 people. About 99 out of 100 people did not have stomach or bowel discomforts from ibuprofen.	For the evaluation of adverse events, the following scheme is used:	
	Very often:	More than 1 patients of 10
	Often:	1 to 10 patients of 100
	Occasional:	1 to 10 patients of 1,000
	Rare:	1 to 10 patients of 10,000
	Very rare:	Less than 1 patient of 10,000
	Unknown:	Frequencies unknown because of availability of data
	Diseases of the gastrointestinal system: Very often: gastrointestinal conditions such as heartburn, stomach ache, nausea, vomiting, flatulence, diarrhoea and obstipation.	
*Serious (rare) adverse events*	*Serious (rare) adverse events*	
Some people reported other problems while taking ibuprofen. There is some evidence suggesting that ibuprofen may be connected to stomach or bowel bleeding, ulcers or perforations, and severe (in rare cases life-threatening) skin reactions. But there are no good studies that provide information on the frequency of these side effects.	Often: gastric/duodenal ulcer (peptic ulcer) under circumstances with bleeding and rupture, stomatitis with ulcer (ulcerative stomatitis), potentiation of ulcerative colitis or Crohn’s disease Occasional: gastritis Very rare: oesophagitis and pancreatitis, intestinal diaphragmatic narrowing	

*Abbreviation*: *PIL* package information leaflet

### Outcomes

Our primary outcome was the nocebo effect. For the quantification of the nocebo effect, we assessed the number of reported AEs. Given that AEs are caused only by pharmacological mechanisms of the medication, there should be no difference between groups that receive different information on AEs.

As secondary outcomes, we analysed self-reported adherence to intake duration and intake frequency as agreed with the treating physician (fully adherent participants versus other). Moreover, we measured the following outcomes in the PIL groups:

- subjective increase of knowledge about effectiveness (question: Do you think the PIL increased your knowledge on the effectiveness of ibuprofen? [yes versus no])
- subjective increase of knowledge about AEs (question: Do you think the PIL increased your knowledge on AEs of ibuprofen? [yes versus no])
- comprehensibility (numeric rating scale [NRS] score of 0–10)
- anxiety regarding AEs provoked by the descriptions in the PIL (NRS score of 0–10).

### Data collection and blinding

We developed questionnaires for standardised data collection. Before surgery, all patients completed a baseline questionnaire. Participants were called 2–3 days after discharge and were interviewed to collect all outcome data. We standardised the interviews by use of a questionnaire and an interviewer guide. The baseline and outcome questionnaires were piloted for comprehensibility with six patients. We asked specifically for AEs known to be caused by ibuprofen (e.g., gastrointestinal or neurological symptoms) to avoid having patients attribute unspecific symptoms (e.g., itch) to the medication.

We called patients up to three times to collect data. When this was unsuccessful, we mailed them an adapted paper version of the questionnaire with the request to send it back within two weeks. If the questionnaire was not sent back in time, participants received a text message on their cell phone with a polite reminder. Patients who did not answer after this last attempt were considered lost to follow-up. All collected data were entered into a standardised case report form (CRF).

Owing to the nature of the intervention, it was not possible to blind participants and personnel that handed out the PILs. However, although the participants were informed that they participated in an intervention study on health information, they were not aware of the exact purpose of the study. The questionnaires used for assessing outcomes in participants who were allocated to one of the PIL groups included more questions (design of the PIL, knowledge of AEs, and anxiety while reading the PIL) than the questionnaire used in patients who received no PIL. Therefore, the study personnel who assessed outcomes knew whether the participants received a PIL but were blinded to the type of PIL.

### Statistical analysis and randomisation

#### Sample size calculation

No similar studies exist so far. Therefore, we planned this study as a pilot study and did not perform a formal sample size calculation. We planned to include at least 20 patients per group to ensure a reliable basis for sample size calculation and we estimated this to be sufficient to identify possible problems with the design or conduct of the study with high confidence [32].

### Randomisation and allocation concealment

We allocated participants to the study groups by using minimisation with a random component (biased coin randomisation) [33]. The factors used for minimisation were age (18–34, 35–65 and >65), gender, outpatient versus inpatient treatment as proxy for type of pain medication regime (only ibuprofen versus ibuprofen plus opioids before discharge), and professional education (university degree versus other). The allocation was performed centrally, shortly before the intervention, to guarantee allocation concealment.

#### Statistical analysis primary outcome

We analysed the influence of the type of information on AEs by using a logistic regression adjusted for the minimisation variables, namely age (<50 versus >50), gender, professional education, and type of pain medication (only ibuprofen versus ibuprofen plus other) and, in addition, for marital status and employment status (employed versus unemployed). We converted odds ratios into relative risk to facilitate interpretation [34].

We performed all analyses of the AEs on an intention-to-treat basis. In the primary analysis, we assumed that all participants with missing data had no AEs (conservative analysis). In addition, we performed a sensitivity analysis by using multiple imputation (Markov chain Monte Carlo; five imputation data sets).

To account for multiplicity, we ordered our hypotheses *a priori* [35]. This means we first tested the overall null hypothesis that there is no difference between at least one of the three groups (H_0_: Simplified-PIL = No-PIL = Standard-PIL; alpha level: 5%). Pairwise confirmatory group comparisons would have been performed only in the case that the overall null hypothesis is rejected.

We conducted a sensitivity analysis by using the same methods described above only for those participants who were allocated to one of the PIL groups and who stated that they have read the PIL. For all group comparisons, we calculated relative risks (RRs) or means with 95% confidence intervals (CIs).

#### Statistical analysis secondary outcomes

We calculated RRs with 95% CIs to analyse adherence. We included only patients in the adherence analysis who were instructed on how to take ibuprofen (duration and intake frequency) and excluded patients who took medication only as needed. In the outcomes used to assess the PILs, we included only patients who read one of the PILs. We performed all analyses on the secondary outcomes according to the randomised allocation but only on patients who had responses in the CRF for the respective outcome; that is, we did not impute missing data.

## Results

### Recruitment

**Fig. 1:**
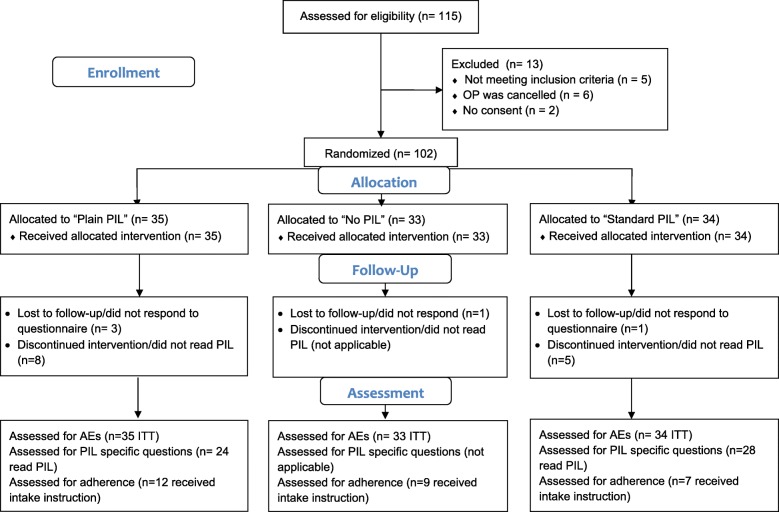
Patient flow diagram. *Abbreviations*: *AE* adverse event, *ITT* intention to treat, *OP* operation, *PIL* package information leaflet

The numbers of participants screened, enrolled and allocated between April and September 2018 are shown in the CONSORT flowchart (Fig. 1). Recruitment was stopped after this pre-specified recruitment period. We randomly assigned 102 patients to one of the three study groups. In total, five participants did not respond to any follow-up attempts and thus were considered lost to follow-up. Complete AE data were available for 95% of patients; 24 and 28 patients read the PIL in the Simplified-PIL and Standard-PIL groups, respectively.

### Baseline characteristics

The baseline characteristics of the included patients are presented in Table 2. All baseline characteristics except for marital status were well balanced.

**Table 2:** Baseline characteristics

	Simplified-PIL	No-PIL	Standard-PIL
Number	35	33	34
Gender: female, n (%)	13 (37.1)	14 (42.4)	11 (32.4)
Age in years, median (IQR)	41 (32–54)	48 (24–55)	44.5 (28.75–53.25)
Marital status: single, n (%)	17 (48.6)	21 (63.6)	18 (52.9)
Professional education: university degree, n (%)	11 (31.1)	11 (33.3)	12 (35.3)
Employment status, n (%)	28 (80)	26 (78.8)	28 (82.4)

*Abbreviations*: *IQR* interquartile range, *PIL* package information leaflet

### Primary outcome

The difference in the overall comparison of the three groups for the total number of AEs was not statistically significant (*P* = 0.47). Therefore, we did not perform pairwise comparisons.

Table 3 shows the absolute and relative risks with 95% CIs of the Simplified-PIL and No-PIL groups compared with the Standard-PIL group for AEs. In the Simplified-PIL group, six (17.1%) patients had an AE. The number of patients with an AE was even smaller in the No-PIL group (*n* = 4, 12.1%) and highest in the Standard-PIL group (*n* = 8, 23.5%). The adjusted RRs were 0.801 (95% CI 0.268–1.894) in the Simplified-PIL group and 0.497 (95% CI 0.138–1.456) in the No-PIL group compared with the Standard-PIL group. Results of the intention-to-treat analysis, sensitivity analysis and unadjusted analysis were similar (Table 3). The relative reduction in AEs was larger in the population who read the PIL (RR 0.461, 95% CI 0.101–1.587).

**Table 3:** Results for ibuprofen-specific adverse events (nocebo effect)

	Number	Adverse events, n (%)	RR (95% CI)
*Intention-to-treat population (conservative), adjusted for minimisation variables*			
Simplified-PIL	35	6 (17.1)	0.801 (0.268–1.894)
No-PIL	33	4 (12.1)	0.497 (0.138–1.456)
Standard-PIL	34	8 (23.5)	Reference
*Intention-to-treat population (multiple imputation), adjusted for minimisation variables*			
Simplified-PIL	35	NA	0.731 (0.250–1.736)
No-PIL	33	NA	0.500 (0.144–1.431)
Standard-PIL	34	NA	Reference
*Intention-to-treat population (conservative), unadjusted*			
Simplified-PIL	35	6 (17.1)	0.728 (0.253–1.714)
No-PIL	33	4 (12.1)	0.515 (0.153–1.439)
Standard-PIL	34	8 (23.5)	Reference
*Population who read PIL, adjusted for minimisation variables*			
Simplified-PIL	24	3 (12.5)	0.461 (0.101–1.587)
Standard-PIL	28	7 (25.0)	Reference

*Abbreviations*: *CI* confidence interval, *NA* not applicable, *PIL* package information leaflet, *RR* relative risk

### Secondary outcomes

The adherence results are shown in Table 4. In the analysis on adherence, only 28 patients could be included because most of the patients took their pain medication only as needed. Adherence was slightly higher in the Simplified-PIL group (RR 1.472, 95% CI 0.435–2.183) and similar (RR 0.963, 95% CI 0.203–1.973) in the No-PIL group compared with the standard-PIL group. The duration of intake was almost identical in all groups (Table 5).

**Table 4:** Results for intake adherence

	Number*	Adherent patients, n (%)	RR (95% CI)
Simplified-PIL	12	9 (75.00)	1.472 (0.435–2.183)
No-PIL	9	4 (55.6)	0.963 (0.203–1.973)
Standard-PIL	7	4 (57.7)	Reference

*Only patients who took medications according to instructions and responded. *Abbreviations*: *CI* confidence interval, *PIL* package information leaflet, *RR* relative risk

**Table 5:** Results for duration of medication intake

	Number*	Mean in days (95% CI)
Simplified-PIL	32	2.44 (1.92–2.96)
No-PIL	31	2.45 (1.94–2.96)
Standard-PIL	33	2.42 (1.89–2.96)

*Only responders. *Abbreviations*: *CI* confidence interval, *PIL* package information leaflet

The results for patient knowledge are shown in Table 6. A few more patients in the Simplified-PIL group (69.6%) than in the Standard-PIL group (57.1%) reported a knowledge increase about effectiveness of ibuprofen after reading. For knowledge on AEs, results were similar for the two PILs (73.9% versus 75.0%). The Simplified-PIL (mean 8.75, 95% CI 8.15–9.36) was considered a little bit more comprehensible and caused less anxiety from AEs (mean 0.875, 95% CI 0.290–1.460) than the Standard-PIL (mean comprehensibility 7.286, 95% CI 6.440–8.130; mean anxiety 2.815, 95% CI 1.940–3.690) as shown in Table 7.

**Table 6:** Results for increase in knowledge about effectiveness and adverse events

	Number*	Patients reporting an increase, n (%)	RR (95% CI)
*Knowledge on effectiveness*			
Simplified-PIL	23	16 (69.6)	1.217 (0.730–1.540)
Standard-PIL	28	16 (57.1)	Reference
*Knowledge on adverse events*			
Simplified-PIL	23	17 (73.9)	0.985 (0.593–1.212)
Standard-PIL	28	21 (75.0)	Reference

*Only responders. *Abbreviations*: *CI* confidence interval, *PIL* package information leaflet, *RR* relative risk

**Table 7:** Results for comprehensibility of the package information leaflet and anxiety of adverse events

	Number*	Mean NRS score 0–10 (95% CI)
*Comprehensibility of the PIL*		
Simplified-PIL	24	8.75 (8.15–9.36)
Standard-PIL	28	7.286 (6.440–8.130)
*Anxiety of adverse events*		
Simplified-PIL	24	0.875 (0.290–1.460)
Standard-PIL	27	2.815 (1.940–3.690)

*Only responders. *Abbreviations*: *CI* confidence interval, *NRS* numeric rating scale, *PIL* package information leaflet

## Discussion

We observed fewer AEs in the Simplified-PIL group compared with the Standard-PIL group. Furthermore, AEs occurred less frequently in the No-PIL than the Simplified-PIL group.

If confirmed in a definitive trial, our data would suggest that not telling patients about potential AEs would be the best option for avoiding the nocebo effect. However, fully omitting information on AEs raises fundamental ethical concerns and may result in legal conflicts (e.g., medication packages without PILs). Moreover, it may interfere with shared decision-making. Focusing on evidence-based information and providing it in a plain and “risk neutral” way seem to be an ethically acceptable compromise and—if our results are confirmed—may reduce the nocebo effect compared with the standard PIL in compliance with EU directives.

Our findings are in accordance with several previous studies on different indications and treatments which suggested that anticipation of negative treatment consequences can lead to AEs [7, 8, 36–40]. However, most previous studies were rather artificial regarding the intervention and setting (e.g., negative versus positive suggestions during an investigator-induced pain stimuli in a laboratory) and thus their applicability to treatment with an active intervention under routine care is questionable. Therefore, we tried to expand this experimental knowledge by comparing a revised simplified PIL with a standard PIL and no written information for AEs on a drug as the PIL represents the most widespread written information on AEs. We found first indications that the nocebo effect might be influenced by the way the information is provided in the PIL and consequently may be a serious problem in routine medical care.

Descriptive data suggest that the Simplified-PIL but not oral information only leads to higher adherence than the Standard-PIL, which is consistent with the observation that the anxiety of suffering AEs was highest in the Standard-PIL group. The finding that expectations of possible negative treatment outcomes lead to non-adherent behaviour is also in accordance with previous studies on this topic [41]. We found a small increase in subjective knowledge about effectiveness in the Simplified-PIL group and, although we provided much less detail on AEs in this group, similar judgements on knowledge on AEs compared with the Standard-PIL group. As the Simplified-PIL received higher comprehensibility ratings, we assume that the reason for these effects on patient knowledge is probably better comprehensibility. This supports our presumption that the design criteria (e.g., less information, bigger font size, and visual presentation of frequencies) used for our PIL are more appropriate to present data in an informative and neutral way than is normally done in EU-standard PILs. Irrespective of the nocebo effect, providing understandable information about possible risks and decreasing the amount of anxiety in patients can be valuable goals in themselves. When all outcomes are considered together, the entire pattern of causes and effects seems consistent with intuitive expectations: an increased comprehensibility and reduction of negative expectations (anxiety) on AEs lead to higher adherence and a reduction of the nocebo effect.

### Generalisability

Our data are limited to one pilot trial in one type of patient (orthopaedic surgery), one setting (secondary care) and one medication (ibuprofen), which limits the generalisability of the results. However, we included a broad participant collective of surgical patients, which is probably quite representative of the general surgical hospital population, and apart from our additional information, we did not change routine care in any way. Moreover, we believe that the nocebo effect likely applies to other conditions though possibly to a different degree.

### Feasibility of a definitive trial and necessary modifications

The Simplified-PIL and even more so the No-PIL showed reductions in AEs, as we anticipated. Also, all other effects showed directions as expected. These observations indicate that a definitive RCT would be worthwhile. Some patients mentioned that the Simplified-PIL lacks information on specific groups (e.g., pregnant women). In the definitive RCT, we will modify the Simplified-PIL by inserting a link or QR code to a website where information on specific groups and other additional information (e.g., description of substance) will be available.

### Limitations

The main limitation of our pRCT is the small sample size. As has to be expected in a pilot trial, the 95% CIs of the effect estimates were wide, indicating uncertainty. However, we believe that the data on the impact of the Simplified-PIL from this pilot trial are sufficient to inform the sample size calculation of a definitive trial [42]. Another limitation resulting from the small sample size is that not all possible confounding variables (e.g., marital status) were well balanced between the groups. This may be also true for factors that are difficult to measure, such as personality traits or additional medication [43]. Therefore, there is a risk of confounding bias. In addition, the inability to blind the intervention is a possible source of bias.

## Conclusions

The primary results of our pRCT and other studies suggest that the way that information about AEs is provided in PILs should carefully consider the risk of possible unintended nocebo responses. A well-powered RCT is needed to confirm the results. This pilot trial shows that a larger definitive trial, which allows firm conclusions, is feasible. If the results are confirmed, a revision of the current practice for designing PILs or even providing no information on request of the patient (informed non-information) might be considered as alternative ways of informing patients about AEs.

## Additional files

Additional file 1: Study protocol. (PDF 1752 kb)

Additional file 2: Changes to the protocol. (DOCX 12 kb)

Additional file 3:  Simplified-PIL. (DOCX 48 kb)

Additional file 4: Standard-PIL. (PDF 253 kb)

## Data Availability

Further data are available by TM on request.

## Abbreviations

AE: Adverse event
CI: Confidence interval
CONSORT: Consolidated Standards of Reporting Trials
CRF: Case report form
NRS: Numeric rating scale
PIL: Package information leaflet
pRCT: Pilot randomised controlled trial
RCT: Randomised controlled trial
RR: Relative risk
