# Supplementary material for: Nocebo effects of a simplified package leaflet compared to unstandardised oral information and a standard package leaflet: a pilot randomised controlled trial
Source: Trials. 2019 Jul 26;20:458. doi: 10.1186/s13063-019-3565-3 (PMC6660653; doi:10.1186/s13063-019-3565-3)
Supplement: Supplementary file 1 — Study protocol. (PDF 1752 kb) [file 13063_2019_3565_MOESM1_ESM.pdf]

Studienprotokoll

Neutral formulierte Packungsbeilage oder ausschließlich mündliche Aufklärung gegenüber Packungsbeilage gemäß EU-Richtlinie: eine randomisiere kontrollierte Pilotstudie zur Analyse des Einflusses auf Nocebo-Effekt und Non-Adhärenz

NEUPANO

Eine monozentrische, dreiarmige, randomisierte, kontrollierte Pilotstudie

Studienakronym:	NEUPANO
Protokollversion:	Entwurfsversion vom 10.01.2018/Finalversion V01.02 vom 12.02.2018

Inhaltsverzeichnis

Allgemeine Informationen 4

1.1 Beteiligte Personen, Institutionen und Gremien 4

1.2 Unterschriften 6

1.4 Synopse 7

Ausschlusskriterien 8

1.5 Ablaufdiagramm 10

1.6 Visitenplan 11

1.7 Verzeichnis der Abkürzungen 12

1. Hintergrund 13

3. Studienziele 14

3.1 Primäres Studienziel und primärer Endpunkt 15

3.2 Sekundäre Studienziele und sekundäre Endpunkte 15

4. Studiendesign und -beschreibung 16

4.1 Art der Studie 16

4.2 Art der Therapiezuordnung 16

4.3 Zahl und Art der Vergleichsgruppen 16

4.4 Patientenrekrutierung 17

4.5 Zeitplan 18

6. Auswahl der Patienten 19

6.1 Einschlusskriterien 19

6.2 Ausschlusskriterien 19

7. Studienablauf 20

7.1 Beschreibung des Studienablaufs 20

9. Biometrie 22

9.1 Endpunkte 22

9.2 Definition von Auswertungskollektiven 22

9.3 Planung des Studienumfanges (Fallzahlplanung) 22

9.4 Statistischer Analyseplan 23

9.4. 1 Basischarakteristika 23

9.4. 2 Statistische Verfahren zur Endpunktauswertung 23

9.4. 3 Subgruppenanalyse 24

10. Datenmanagement 25

10.1 Patientenidentifikationsliste 25

10.2 Datenerhebung/Dokumentationsbögen 25
10.3 Studienunterlagen und deren Aufbewahrung (Archivierung) 25
10.4 Datenschutz 25
12. Ethische Belange, gesetzliche und administrative Regelungen 26
12.1 Deklaration von Helsinki und Gute klinische Praxis 26
12.2 Ethik-Kommissionen 26
12.3 Nachträgliche Änderungen 26
12.6 Registrierung 26
12.7 Finanzierung 26
12.8 Abschlussbericht und Publikation 27
13. Literatur 28
14. Anlagen 31
A Case Report Form 31
B Ethikvotum 40

Allgemeine Informationen

1.1 Beteiligte Personen, Institutionen und Gremien

**Studienleiter**<br><br>*Name: Tim Mathes*<br>*Adresse: Institut für Forschung in der Operativen Medizin (IFOM), Ostmerheimer Str. 200, 51109 Köln*<br>*Tel.: +49 (0)221 98957-43*<br>*E-Mail: tim.nathes@uni-wh.de*	**Biometriker**<br><br>*Name: Tim Mathes*<br>*Adresse: Institut für Forschung in der Operativen Medizin (IFOM), Ostmerheimer Str. 200, 51109 Köln*<br>*Tel.: +49 (0)221 98957-43*<br>*E-Mail: tim.nathes@uni-wh.de*
**Stellvertreter des Studienleiters bzw. Mitglieder der Studienleitung**<br>*Name: Barbara Prediger*<br>*Adresse: Institut für Forschung in der Operativen Medizin (IFOM), Ostmerheimer Str. 200, 51109 Köln Tel.: +49 (0)221 98957-42*<br>*E-Mail: barbara.prediger@uni-wh.de*	**Monitoring**<br>*Name: Barbara Prediger*<br>*Adresse: Institut für Forschung in der Operativen Medizin (IFOM), Ostmerheimer Str. 200, 51109 Köln*<br>*Tel.: +49 (0)221 98957-42*<br>*E-Mail: barbara.prediger@uni-wh.de*<br><br>*Name: Esther Meyer*<br>*Adresse: Ostmerheimer Str. 200 51109*<br>*Tel.: +49 (0)221 98957-44*<br>*E-Mail: esther.meyer@uni-wh.de*
**Datenmanagement**<br>*Name: Barbara Prediger*<br>*Adresse: Ostmerheimer Str. 200, 51109*<br>*Tel.: +49 (0)221 98957-42*<br>*E-Mail: barbara.prediger@uni-wh.de*	**Studienkoordination/Projektmanagement**<br><br>*Institut für Forschung in der Operativen Medizin (IFOM), Ostmerheimer Str. 200, 51109 Köln*

Name: Esther Meyer<br>*Adresse: Ostmerheimer Str. 200, 51109*<br>*Tel.: +49 (0)221 98957-44*<br>*E-Mail: esther.meyer@uni-wh.de*	

1.2 Unterschriften

____

Name, Studienleiter Datum

____

Name, Stellvertreter des Studienleiters Datum

____

Name, Biometriker Datum

1.4 Synopse

**Titel der Studie**	Neutral formulierte Packungsbeilage oder ausschließlich mündliche Aufklärung gegenüber Packungsbeilage gemäß EU-Richtlinie: eine randomisiere kontrollierte Pilotstudie zur Analyse des Einflusses auf Nocebo-Effekt und Non-Adhärenz
**Kurzbezeichnung der Studie (Akronym)**	Einfluss einer neutral formulierten Packungsbeilage auf den Noceboeffekt eine randomisierte, kontrollierte Studie (NEUPANO)
**Studienleiter**	Tim Mathes
**Stellvertreter des Studienleiters**	Barbara Prediger
**Indikation/Zielpopulation/ Erkrankung**	- Orthopädische Operation<br>- Einnahme von Ibuprofen
**Studiendesign/Methodik**	- Studiendesign: monozentrische, dreiarmige, kontrollierte, randomisierte Pilotstudie<br>- Verblindung: auf Grund der Art der Intervention ist eine Verblindung der Intervention nicht möglich. Die Erfassung der Endpunkte erfolgt verblindet<br>- Randomisierung: zentralisiert, Minimization
**Ziele der klinischen Prüfung/Zielstellung**	Primäres Ziel: Ziel dieser randomisierten kontrollierten Pilotstudie ist es den Einfluss unterschiedlicher Aufklärung über Nebenwirkungen auf die Häufigkeit und Intensität tatsächlich aufgetretener Nebenwirkungen zu analysieren<br><br>Sekundäre Ziele: Erfassung der Non-Adhärenz, durch Nebenwirkungen bedingten Ressourcenverbrauchs und Zufriedenheit der Patienten mit der Aufklärung
**Zielgrößen/-kriterien/ Endpunkte**	Primäre Zielgröße/Hauptzielkriterium: Anzahl an, durch den Patienten berichtetet (wahrgenommene), unerwünschten Ereignissen<br><br>Sekundäre Zielgrößen/Nebenzielkriterien: Adhärenz, Zufriedenheit der Patienten mit der Packungsbeilage, Ressourcenverbrauch
**Patientenzahl**	Randomisiert: min. 69<br>Anzahl in Analyse: 60

**Einschlusskriterien**	Es werden Patienten in die Studie eingeschlossen, die die folgenden Einschlusskriterien erfüllen:<br>• Orthopädischer elektiver Eingriff<br>• Alter ≥18<br>• Einnahme von oraler postoperativer Schmerzmedikation (Ibuprofen 600)<br>• Postoperative Schmerzmedikation anders als Ibuprofen 600 (Opioide, Opiate, Paracetamol) nicht länger als 1-2 Tage postop., bzw. nicht bei Entlassung<br>• Deutsche Sprachkenntnisse<br>• Keine kognitiven Defizite<br>• Schriftlichen Einverständniserklärung ICH GCP (E6) [1]
**Ausschlusskriterien**	Ausschlusskriterien<br>Patienten die die folgenden Kriterien aufweisen werden ausgeschlossen:<br>• Einnahme von Schmerzmitteln vor der Operation zur Behandlung chronischer Schmerzen<br>• Einnahme von anderen Medikamenten die ähnliche Nebenwirkungen haben<br>• Schwerwiegende Komorbidität oder Erkrankungen, die ähnliche Symptome hervorrufen, wie potentielle Nebenwirkungen (Einschätzung des Arztes)<br>• Polytrauma<br>• Stationäre Reha voraussichtlich >eine Woche nach Krankenhausentlassung
**Behandlungen/Verfahren, Behandlungsplan (inkl. Nachsorge)**	***Intervention***<br>- Modifizierte Packungsbeilage: Simplifizierte und auf Vermeidung von Risikoverzerrung ausgelegte Packungsbeilage zur Aufklärung über Nebenwirkungen der postoperativen Schmerzmedikamente. Die Darstellungen und Formulierungen der Packungsbeilage orientieren sich an den Erkenntnissen aus Forschung zur evidenzbasierten Patienteninformationen und Risikokommunikation. Es werden in dieser die gleichen Inhalte aufgenommen, wie in der gesetzlich vorgeschriebenen Packungsbeilage, um zu gewährleisten, dass der Inhalt der Packungsbeilage den gesetzlichen Anforderungen entspricht.<br>- Routine Versorgung im Krankenhaus: ggf. mündliche Aufklärung über mögliche Nebenwirkungen: Der Patient wird ausschließlich mündlich aufgeklärt und erhält keine Packungsbeilage.

	Kontrolle<br>Packungsbeilage gemäß der EU-Richtlinie 2001/83/EG (Standardpackungsbeilage).
**Zeitplan (Studiendauer)**	Patientenbezogen: durchschnittlich ca. 6-9 Tage, min. 2 Tage,<br>max. 6 Wochen Nachbeobachtungsdauer: ca. 2-3 Tage nach Krankenhausentlassung<br>Studienbezogen:<br>Beginn (Zeitpunkt): 01.04.2018<br>Rekrutierungsdauer: 10 Wochen<br>Gesamtdauer: 3 Monate<br>voraussichtlicher Abschluss einschl. Auswertung (Zeitpunkt): 31.10.2018
**Statistische Methoden**	Zur Prüfung auf statistisch signifikante Unterschiede der berichteten unerwünschten Ereignisse (primärer Endpunkt) zwischen den Studiengruppen wird eine Mean cumulative function (MCF), verwendet.<br>Zur Berücksichtigung der Multiplizität aufgrund des Vergleiches von mehr als zwei Gruppen werden für alle statistischen Vergleiche auf signifikante Unterschiede die Hypothesen a-priori geordnet.
**Finanzierung**	Deutsche Forschungsgemeinschaft (DFG)

1.5 Ablaufdiagramm

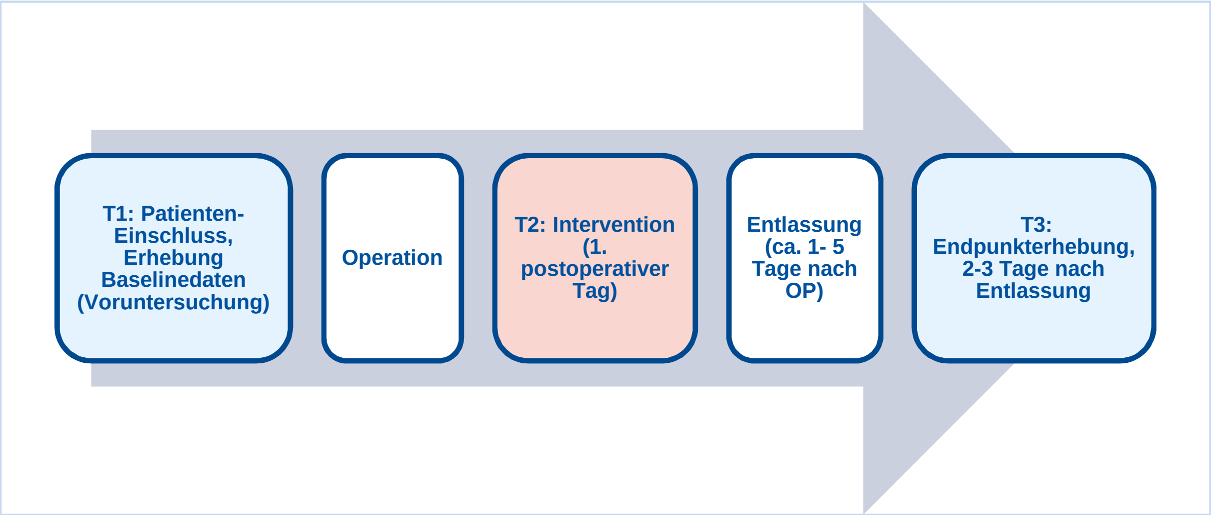

1.6 Visitenplan

	T1	T2	T3
Basisdatenerhebung	X		
Aufklärung/Einwilligung	X		
Ein- und Ausschlusskriterien	X		
Randomisierung		X	
Erbringung der Intervention		X	
Erfassung Begleitmedikation			
Erfassung unerwünschter Ereignisse			X
Erfassung Patientenzufriedenheit und Verständlichkeit			X
Erfassung Ressourcenverbrauch			X

1.7 Verzeichnis der Abkürzungen

VAS	Visuelle Analogskala
ITT	Intention-To-Treat-Prinzip
MCF	Mean cumulative function
ANOVA	Analysis of Variance
RR	Relatives Risiko
p-CRF	paper based case report form
TIDieR	Better reporting of interventions: template for intervention description and replication

1. Hintergrund

Der Nocebo-Effekt bezeichnet den Zusammenhang der Erwartungshaltung bezüglich negativer Wirkungen und dem tatsächlichen Auftreten von negativen Symptomen [2]. Diese negativen Wirkungen einer Intervention sind also nicht pharmakologisch begründet, sondern sie werden, analog zum Placeboeffekt, durch den psychosozialen Kontext, insbesondere durch Suggestion und Konditionierung hervorgerufen. Im Gegensatz zum Placebo-Effekt ist der Nocebo-Effekt negativ gerichtet, d.h. er bezeichnet das zusätzliche Auftreten und die Verstärkung von (unspezifischen) Symptomen und nicht die Verbesserung des Gesundheitszustandes. Bezogen auf unerwünschte Ereignisse von Medikamenten ist es somit der Anteil an Nebenwirkungen, der zusätzlich zu den pharmakologischen, kausal zu erklärenden, unerwünschten Ereignissen (unerwünschte Arzneimittelwirkungen) auftritt.

Der Nocebo-Effekt ist erstmalig aufgrund der Beobachtung in den Fokus des Forschungsinteresses getreten, dass auch von ausschließlich mit Placebo behandelten Patienten in randomisierten klinischen Studien häufig Nebenwirkungssymptome berichtet wurden [3]. Der Nocebo-Effekt ist aus vielfältigen Gründen patientenrelevant. Neben der direkten Reduktion der Lebensqualität durch zusätzliche Nebenwirkungen, weisen Studienergebnisse darauf hin, dass der Nocebo-Effekt auch eine negative Wirkung auf die Wirksamkeit der Therapie selbst haben kann [4]. Darüber hinaus sind die negativen Erwartungen sowie das Auftreten von Nebenwirkungen bei vielen Erkrankungen einer der Hauptfaktoren für eine nicht korrekte Einnahme der Medikamente (Non-Adhärenz), insbesondere für einen frühzeitigen Therapieabbruch [5, 6]. Non-Adhärenz kann wiederrum einen negativen Einfluss auf die Therapiewirksamkeit nach sich ziehen. Ferner kann schon alleine die Angst vor Nebenwirkungen dazu führen, dass Patienten mit einer Therapie nicht beginnen [7, 8]. Neben den Folgen für den Patienten selbst, können darüber hinaus, durch zusätzliche Arztkontakte oder zusätzliche Medikamenteneinnahme anlässlich negativer Symptome, finanzielle Belastungen für das Gesundheitssystem entstehen [8].

In der weiter vorangeschrittenen Placeboforschung hat sich gezeigt, dass die Effektgrößen im, ausschließlich mit Placebo behandelten, Studienarm oftmals vergleichbar sind mit den Effektgrößen im aktiven Behandlungsarm [9]. Unter der Annahme, dass beiden Phänomenen (Nocebo, Placebo) sehr ähnliche psychologische und neurobiologische Mechanismen zugrunde liegen, kann vermutet werden, dass auch Nebenwirkungen zu einem Großteil einer nicht pharmakologischen Ursache unterliegen [10]. Diese Vermutung wurde später in Reviews mit dem Ziel der Quantifizierung des Nocebo-Effekts systematisch analysiert und für verschiedene Indikationen bestätigt. Es können auch in Placebogruppen häufig in erheblichem, mitunter gleichem Maße, Nebenwirkungen, wie im aktiven Behandlungsarm nachgewiesen, auftreten [11-14].

Bisherige Forschung untersucht zumeist den experimentellen Nocebo-Effekt, der durch verbale Suggestion in einer aktiven Behandlung entsteht. Diese Studien kommen zu dem Ergebnis, dass der Nocebo-Effekt durch Kommunikation beeinflusst werden kann. Sie belegen, dass unter aktiver Behandlung vermehrt negative Wirkungen (z.B. Schmerz) von den Patienten berichtet werden, wenn zuvor eine verbale Suggestion stattgefunden hat [15, 16]. Auch in Studien, die den Einfluss von verbaler Aufklärung gegenüber keiner verbalen Aufklärung über negative Wirkungen unter aktiver Behandlung vergleichen, zeigte sich ein signifikanter Einfluss der Aufklärung auf die Anzahl an

berichteten negativen Wirkungen [17-19]. Zudem weisen Studien auf eine Beeinflussbarkeit des Nocebo-Effekts durch die Art der schriftlichen Aufklärung hin [20, 21].

Bisher finden sich jedoch keine quantitativen Studien zum tatsächlichen Einfluss von unterschiedlich ausgestalteten, schriftlichen Aufklärungen auf die Stärke des Nocebo-Effekts. Darüber hinaus wurden bisher nicht die Unterschiede von mündlicher gegenüber schriftlicher Aufklärung über Nebenwirkungen auf die berichtete Anzahl von negativen Symptomen untersucht.

Die im Folgenden beschriebene Studie soll diese zwei Wissenslücken, bezüglich des, durch schriftliche Kommunikation, induzierten Nocebo-Effektes bei Patienten die aktiv behandelt werden, schließen.

Diese Forschungsfragen sind insbesondere von Bedeutung, da aus dem Bereich der Kommunikationswissenschaften bekannt ist, dass unterschiedliche Risikokommunikation mit unterschiedlicher Risikoeinschätzung bzw. einer Verzerrung der Risikoeinschätzung assoziiert sein kann. Der Nocebo-Effekt wiederum wird insbesondere durch die (Risiko-) Erwartungen des Patienten beeinflusst [22]. Dabei deuten Studienergebnisse darauf hin, dass Konditionierung nur einen geringen Einfluss auf den Nocebo-Effekt zu haben scheint [6]. Diese Beobachtung legt nahe, dass ein Großteil von Nebenwirkungen, durch Suggestion bedingt ist und somit auch manipuliert werden kann.

3. Studienziele

Ziel dieser randomisierten kontrollierten Pilotstudie ist es, den Einfluss von schriftlicher Aufklärung (Packungsbeilagen) über mögliche Nebenwirkungen eines Arzneimittels auf die Häufigkeit des Auftretens von Nebenwirkungen und die Adhärenz zu analysieren.

Hierdurch soll zum einen untersucht werden, ob unterschiedliche Darstellungen, Formulierungen etc. von schriftlicher Aufklärung zu Nebenwirkungen einen Einfluss auf die Häufigkeit der (wahrgenommenen) Nebenwirkungen und auf die Adhärenz haben (a). Zum anderen soll der durch schriftliche Suggestion verursachte Anteil von Nebenwirkungen und das Ausmaß der Non-Adhärenz quantifiziert werden (b).

Durch die Ermittlung des Unterschiedes an berichteten Nebenwirkungen zwischen den aktiven Behandlungsgruppen, in denen lediglich die Kommunikation von Nebenwirkungen unterschiedlich ist bzw. nicht stattfindet, lässt sich eine Aussage über den, durch Kommunikation verursachten, Anteil an Nebenwirkungen und den Anteil von Non-Adhärenz treffen. Hieraus lässt sich eine Schlussfolgerungen zur Beeinflussbarkeit des Nocebo-Effektes bei der Einnahme einer aktiven Therapie ziehen. Mit der Studie soll, auf diesem Weg, überprüft werden, ob ein bestimmter Anteil an Nebenwirkungen und Non-Adhärenz in der medizinischen Routineversorgung, durch modifizierte Darstellung oder der Möglichkeit des Verzichts auf schriftliche Aufklärung über Nebenwirkungen, vermieden werden könnte.

Bisher wurde die Nocebo-Problematik nicht in der Arzneimittelregulation berücksichtigt. Im Gegenteil, werden in Packungsbeilagen alle bekannt gewordenen Nebenwirkungen angegeben, um Haftungsfälle für die Hersteller auszuschließen.

Zur Erhöhung der Relevanz und Übertragbarkeit der Ergebnisse setzt die untersuchte Intervention aus diesem Grund bei den Packungsbeilagen an, da diese die weitverbreitetste Form der Aufklärung von Nebenwirkungen sind. Aus Versorgungsforschungssicht ist diese Fragestellung von großer Bedeutung, da Packungsbeilagen bei der Einnahme jeglicher Arzneimittel beigefügt werden.

Die Erkenntnisse der geplanten Studie können einen Beitrag leisten, die schriftliche Darstellung von Nebenwirkungen in Packungsbeilagen dahingehend zu verbessern, dass die Verständlichkeit erhöht und negative Erwartungshaltungen minimiert und somit die assoziierten Gefahren von zusätzlichen, vermeidbaren Nebenwirkungen, mangelnder Adhärenz und eingeschränkter Wirksamkeit (durch Non-Adhärenz) reduziert werden. Darüber hinaus können die Ergebnisse darauf hinweisen, dass Patienten vorab über die Konsequenz der Konfrontation mit möglichen Nebenwirkungen - dem erhöhtem Risiko von Nebenwirkungen - aufgeklärt werden sollten [23].

3.1 Primäres Studienziel und primärer Endpunkt

Der primäre Endpunkt ist die Häufigkeit der, durch den Patienten, berichteten (wahrgenommenen) Nebenwirkungen.

3.2 Sekundäre Studienziele und sekundäre Endpunkte

Als sekundärer Endpunkt wird die Patientenadhärenz ausgewertet.

Weiterhin wird die Verständlichkeit und Zufriedenheit mit den Aufklärungen über Nebenwirkungen, die Angst vor Nebenwirkungen und der Einflusses auf den Wissensstand der Patienten/innen, erfasst.

Zur Quantifizierung der ökonomischen Auswirkungen des Nocebo-Effektes wird der, durch Nebenwirkungen entstandene, Ressourcenverbrauch erfasst.

4. Studiendesign und -beschreibung

4.1 Art der Studie

Es handelt sich um eine monozentrische, dreiarmige, vergleichende, randomisierte, kontrollierte Pilotstudie.

4.2 Art der Therapiezuordnung

Für die Zuweisung der Patienten zu den Studiengruppen wird das Verfahren der Minimisierung mit einer Zufallskomponente verwendet (Minimization with Randomization) [24]. Die Minimisierung erfolgt anhand von Alter, Geschlecht, Bildungsstatus und geplanter Schmerzmedikation (Ibuprofen 600 vs Ibuprofen 600 + Anderes) [25].

Die Allokation wird mittels Computer generiert. Die Patienten werden in einem Verhältnis von 1:1:1 der jeweiligen Gruppe zugeteilt. Die Zuteilung erfolgt zentralisiert, um zu vermeiden, dass die Zuteilung vorhergesehen werden kann. Dies wird dadurch sichergestellt, dass dem Prüfer erst nach der Einwilligung der Patienten die Zuteilung mitgeteilt wird.

4.3 Zahl und Art der Vergleichsgruppen

Es werden drei parallele Gruppen gebildet. Zwei Interventionsgruppen und eine Kontrollgruppe.

In der ersten Gruppe erhalten alle Patienten, neben der im Krankenhaus üblichen Aufklärung, eine simplifizierte und auf Vermeidung von verzerrter Risikowahrnehmung ausgelegte Packungsbeilage (neutrale Packungsbeilage) zur Aufklärung über Nebenwirkungen der Medikamente für die postoperative Schmerzmedikation (Ibuprofen, Interventionsgruppe a).

In der neutralen Packungsbeilage werden die gleichen Inhalte (z.B. Wirkung, Kontraindikationen, behandlungsbedürftige Nebenwirkungen, häufige Nebenwirkungen) aufgenommen wie in der gesetzlich vorgeschriebenen Packungsbeilage, um zu gewährleisten, dass der Inhalt der neu entwickelten Packungsbeilage prinzipiell den gesetzlichen Anforderungen entspricht. Allerdings werden lediglich unerwünschte Ereignisse dargestellt, die empirisch belegt, die sehr häufig auftreten (>10%), und Behandlungsbedürftige unerwünscht Ereignisse sowie schwerwiegende unerwünschte Ereignisse darstellen. Darüber hinaus müssen die Nebenwirkungen durch qualitative hochwertige kontrollierte Studien – wenn möglich RCT oder Systematische Reviews von RCT - empirisch belegt sein. Gleichermaßen wird für die Kontraindikationen vorgegangen.

Die Darstellungen und Formulierungen orientieren sich an den Erkenntnissen zu evidenzbasierten Patienteninformationen und systematischen Reviews zur Risikokommunikation (vgl. z.B. Ancker et al. 2006 [26] und Trevena et al. 2006 [27]).

Zur Vermeidung von Risikoverzerrung werden die Angaben zu Häufigkeiten ausschließlich direkt beziffert, um die Gefahr von Überschätzung des Risikos durch die Verwendung verbal beschreibender Kategorien (z.B. sehr häufig) zu reduzieren [28]. Es wird nur die Differenz an Nebenwirkungen

gegenüber Placebo dargestellt, da nur diese als tatsächlich durch den Wirkstoff verursacht angesehen werden können.

Da durch die numerische Angabe anstatt von verbaler Kategorien u.U. die Verständlichkeit reduziert wird, werden die Häufigkeiten prozentual und als Ereignisrate (z.B. 1 von 10, 1 von 100) angeben sowie zusätzlich mit Hilfe von Piktogrammen verdeutlicht [29]. Wichtige Informationen werden farblich hervorgehoben. Die Angaben zu Nebenwirkungen werden auf Verträglichkeit fokussiert formuliert (Gain-Framing). Auf Verträglichkeit fokussiert bedeutet hierbei, dass nicht der Anteil an Behandelten angegeben wird, die von den jeweiligen Nebenwirkungen betroffen sind, sondern der Anteil an Personen, die nicht von den Nebenwirkungen betroffen sind. Zudem wird auf eine einfache und allgemein verständliche Formulierung geachtet. Beispielsweise werden Fremdwörter übersetzt und um die Gesamtverständlichkeit zu erhöhen, nötigenfalls Beispiele und Erklärungen ergänzt. Darüber hinaus werden die Informationen zusammenfassend dargestellt und der therapeutische Effekt beschrieben [4, 30]. Weiterhin wird versucht, den Nutzen der Einnahme hervorzuheben (Gain-Framing) und eine möglichst einfache Sprache zu verwenden (z.B. Vermeidung von unnötigen Fachtermini). Um die Konsequenzen der Nebenwirkungen besser einschätzen zu können, wird die voraussichtliche Dauer (z.B. legt sich in der Regel innerhalb von drei bis vier Tagen wieder) und der Schweregrad vermittelt (z.B. nur einer von 100 Personen hat auf Grund von Bauchschmerzen die Studie abgebrochen). Falls die Nebenwirkungen nicht hinreichend belegt sind, (z.B. Häufigkeit Nebenwirkungen versus Placebo p-Wert >0,2) wird die Unsicherheit der Aussage zum Ausdruck gebracht (z.B. es gibt Hinweise).

Die Packungsbeilage wird von einem Team aus Wissenschaftlern mit Erfahrung in Evidenzbasierter Medizin, Patientenkommunikation und Pharmakologie entwickelt.

Das Studienpersonal bittet die Patienten, die Packungsbeilage durchzulesen.

Es wird ein Pretest mit etwa 6 Patienten für die neu entwickelte Packungsbeilage vorgenommen, um Verständlichkeit, emotionale Reaktionen, Aufbau etc. zu testen. Hierzu werden die Patienten zufällig ausgewählt.

In der zweiten Interventionsgruppe erhalten die Patienten ggf. ausschließlich eine mündliche Aufklärung entsprechend dem üblichen Vorgehen im Krankenhaus (mündliche Aufklärung).

In der Kontrollgruppe erhält jeder Patient, neben der im Krankenhaus üblichen Aufklärung, die zum jeweiligen Präparat vom Hersteller beigefügte Packungsbeilage für die postoperative Schmerzmedikation (Ibuprofen), gemäß der EU-Richtlinie 2001/83/EG (Standard-Packungsbeilage, Kontrollgruppe). Auch hier bittet der Arzt die Patienten die Packungsbeilage durchzulesen.

4.4 Patientenrekrutierung

Es werden alle Patienten eingeschlossen, die die unten genannten Einschlusskriterien erfüllen. Elektive Eingriffe werden im Studienzentrum, in der Regel von Montag bis Freitag, durchgeführt.

Unter Berücksichtigung der jährlichen Fallzahl, der Einschlusskriterien und der Einwilligungsquote (Annahme 80%), wird davon ausgegangen, dass mindestens 2 Patienten pro Tag rekrutiert werden können. Es sollen mindestens 69 Patienten eingeschlossen werden. Bei einer durchschnittlichen Rekrutierungsrate von 2 Patienten pro Tag und 21 Werktagen im Monat ergibt sich hieraus ein Rekrutierungszeitraum von mindestens 1,5 Monaten.

Die Patienten werden an der Klinik für Orthopädie, Unfallchirurgie und Sporttraumatologie des Krankenhauses Köln Merheim, Kliniken der Stadt Köln gGmbH rekrutiert.

4.5 Zeitplan

		**Monat**									
**Arbeitsschritt**		**1**	**2**	**3**	**4**	**5**	**6**	**7**	**8**	**9**	**10**
	Verfassung Studienprotokoll	■									
	Erstellung neutrale Packungsbeilage und Studiematerialien (z.B. CRF)		■	■							
	Rekrutierung				■	■					
	Nachbeobachtung					■	■				
	Dateneingabe/Kontrolle						■	■			
	Auswertung								■	■	
	Verfassen Publikation und Abschlussbericht									■	■

Die erwartete Gesamtstudiendauer der Studie beträgt 10 Monate. Abbildung 1 eins zeigt den Zeitplan.

Die Patienten haben nach der Operation eine Verweildauer von ca. 3-5 Tage. Die Erfassung der Endpunkte soll innerhalb von zwei bis drei Tagen nach der Krankenhausentlassung stattfinden. Somit beträgt die Studiendauer für jeden Patient etwa neun Tage. Diese kann allerdings, insbesondere bei längerer oder kürzerer Krankenhausverweildauer und nicht Erreichen bei Erstversuch für die Endpunkterfassung (z.B. nicht telefonisch erreichbar), abweichen.

6. Auswahl der Patienten

6.1 Einschlusskriterien

Es werden Patienten in die Studie eingeschlossen, die die folgenden Einschlusskriterien erfüllen:

- Orthopädischer elektiver Eingriff
- Alter ≥18
- Einnahme von oraler postoperativer Schmerzmedikation (Ibuprofen 600)
- Postoperative Schmerzmedikation anders als Ibuprofen 600 (Opioide, Opiate, Paracetamol) nicht länger als 1-2 Tage postop., bzw. nicht bei Entlassung
- Deutsche Sprachkenntnisse
- Keine kognitiven Defizite
- Schriftlichen Einverständniserklärung ICH GCP (E6) [1]

6.2 Ausschlusskriterien

Patienten die die folgenden Kriterien aufweisen werden ausgeschlossen:

- Einnahme von Schmerzmitteln vor der Operation zur Behandlung chronischer Schmerzen
- Einnahme von anderen Medikamenten die ähnliche Nebenwirkungen haben
- Schwerwiegende Komorbidität oder Erkrankungen, die ähnliche Symptome hervorrufen, wie potentielle Nebenwirkungen (Einschätzung des Arztes)
- Polytrauma
- Stationäre Reha voraussichtlich >eine Woche nach Krankenhausentlassung

Darüber hinaus werden keine Ausschlusskriterien definiert. Dies dient zum einen der Erhöhung der externen Validität. Zum anderen ist bisher nicht bekannt, welche Faktoren den Nocebo-Effekt beeinflussen [6]. Eine Definition von Ausschlusskriterien müsste auf Basis von Annahmen getroffen werden und würde folglich nur, falls zufällig die „richtigen“ Faktoren gewählt werden, zu einer relevanten Steigerung der internen Validität und Varianzreduktion beitragen.

7. Studienablauf

Die Anfrage zur Teilnahmebereitschaft findet im Rahmen des Aufklärungsgespräches zur Operation statt. Bei Einwilligung zur Studienteilnahme werden die demografischen und andere Baselinedaten (z.B. Indikation, vorgesehene Dosierung von Ibuprofen) durch den Prüfer dem Krankenhausinformationssystem entnommen oder, falls nicht in diesem enthalten, die Patient mittels eines Fragebogens befragt (z.B. Bildungsstand, Einkommen).

Die Randomisierung findet zentralisiert, zwischen dem chirurgischen Eingriff und vor Gabe der ersten postoperativen Schmerzmedikation, statt. Die jeweilige Packungsbeilage wird den Patienten vom Arzt, Pflegepersonal oder Studienpersonal, am ersten postoperativen Tag, ausgeteilt.

Die Patienten werden zur Erfassung der Nebenwirkungen gebeten, zu den in der Packungsbeilage genannten Nebenwirkungen jeweils die Häufigkeiten des Auftretens anzugeben und ggf. die Intensität (z.B. Bauchschmerzen) mittels visueller Analogskala (VAS). Es werden subjektive (z.B. Übelkeit) und objektive Endpunkte (z.B. Hautausschlag) unterschieden.

Als sekundärer Endpunkt wird die Patientenadhärenz ausgewertet.

Für die Patienten in den Gruppen, die eine Packungsbeilage erhalten, wird weiterhin ihre Meinung bezüglich Verständlichkeit, Zufriedenheit, Angst vor Nebenwirkungen sowie Wissensstand der Patienten erfasst [8].

Zur Quantifizierung der ökonomischen Auswirkungen des Nocebo-Effektes wird der durch Nebenwirkungen entstandene Ressourcenverbrauch erfasst. Hierzu werden zum einen die durch Nebenwirkungen verursachten Arzt-Patientenkontakte und zum anderen die zusätzlich eingenommenen Arzneimittel abgefragt. Zur Messung werden die Patienten gebeten, anzugeben wie häufig sie einen Arzt aufgrund von Nebenwirkungen aufgesucht und/oder weitere Arzneimittel eingenommen haben.

Zur Bestimmung der Interventionsintegrität wird geprüft, ob die Teilnehmer die Packungsbeilage gelesen haben.

Für sämtliche Erhebungen der Endpunkte werden die Patienten durch das Studienpersonal telefonisch befragt. Die telefonische Befragung findet am 2. oder 3. Tag nach der Krankenhausentlassung statt. Falls ein Patient nicht telefonisch erreicht werden kann, wird ihm postalisch der Fragebogen zugesendet. Im Falle, dass die Rücksendung des Fragebogens nicht innerhalb einer Woche erfolgt ist, wird eine SMS zur Erinnerung versendet. Patienten, bei denen eine Nachuntersuchung in Krankenhaus geplant ist, werden, falls sie nicht zuvor telefonisch erreicht wurden, im Rahmen dieser befragt.

7.1 Beschreibung des Studienablaufs

Die Anfrage zur Teilnahmebereitschaft und Aufklärung über die Studie findet im Rahmen des Aufklärungsgespräches zur Operation statt. Die Aufklärung jedes Patienten über Wesen, Bedeutung, Ziele, mögliche Risiken, erwartete Vorteile, Tragweite und sonstige Aspekte der Studie erfolgt durch ein Gespräch zwischen Prüfer und dem Patienten. Der Patient erhält die schriftliche

Patienteninformation. Der Prüfer überzeugt sich davon, dass die Aufklärung vom Patienten verstanden wurde. Nach der Aufklärung erhält jeder Patient ausreichend Zeit und Gelegenheit, offene Fragen zu klären und über seine Teilnahme zu entscheiden.

Jeder Patient unterzeichnet seine Einwilligung in die Teilnahme an der Studie eigenhändig schriftlich auf der Einwilligungserklärung [1]. Die Einwilligung des Patienten umfasst auch eine Aufklärung über die Erhebung und Verarbeitung von personenbezogenen Daten. Deshalb werden die Patienten explizit über Zweck und Umfang der Erhebung und die Verwendung dieser Daten, insbesondere von Gesundheitsdaten, informiert.

Es werden nur Patienten eingeschlossen, die die Einwilligungserklärung selbständig unterzeichnen können. Ein Exemplar der unterschriebenen Einwilligungserklärung (Kopie) wird dem Patienten ausgehändigt, das andere verbleibt im Prüfzentrum.

Der Patient kann jederzeit und ohne Angabe von Gründen die Einwilligung zurückziehen und die Behandlung abbrechen bzw. die Studie abbrechen. Der Patient wird in solchen Fällen gebeten, den Abbruchgrund (für die Behandlung oder die Teilnahme) zu nennen. Der Zeitpunkt der Rücknahme der Einwilligung zu Behandlung oder Studie ist zu dokumentieren.

9. Biometrie

9.1 Endpunkte

Es werden die folgenden Endpunkte ausgewertet:

- Primär
    - Rate an berichteten unerwünschten Ereignissen. Diese werden insgesamt betrachtet sowie in subjektiv und objektiv unterschieden. Weiterhin erfolgt eine separate Betrachtung gemäß schwergrad (schwerwiegend, nicht schwerwiegend)
- Sekundär
    - Durchschnittliche Adhärenz**.** Die verschiedenen Adhärenzformen (korrekte Einnahme während des vorgesehenen Einnahmeintervalls, Therapieabbruch). Als nichtadhärent werden Patienten eingestuft, die entweder der Ibuprofen vorzeitig absetzen und/oder angeben, dieses nicht immer korrekt eingenommen zu haben.
    - Durchschnittliche Angst (arithmetisches Mittel)
    - Anteil an Patienten mit gesteigertem Ressourcenverbrauch. Dieses umfasst Patienten mit Arzt-Patienten-Kontakten (Hausarzt, Facharzt, Krankenhaus) und Einnahme von Arzneimittel auf Grund von Nebenwirkungen.

9.2 Definition von Auswertungskollektiven

Die gesamte Analyse wird nach dem Intention-To-Treat-Prinzip (ITT) durchgeführt. In diese Analyse gehen alle Patienten ein, die randomisiert wurden und die Packungsbeilage erhalten haben.
Fehlende Werte werden mittels multipler Imputation ersetzt. Hierfür wird das Markov-Chain-Monte-Carlo-Verfahren angewendet [31].

Es werden zwei ergänzende Analyse durchgeführt. Die erste umfasst ausschließlich Patienten, die angeben, dass sie die Packungsbeilage gelesen haben. Für die zweite ergänzende Analyse werden Patienten ausgeschlossen, die nach der Operation auch andere Schmerzmittel eingenommen haben.

9.3 Planung des Studienumfanges (Fallzahlplanung)

Da bisher keine Studie mit der gleichen Fragestellung durchgeführt wurde und somit in der Literatur keine aussagekräftigen Zahlen zum Unterschied der Häufigkeit von Nebenwirkungen bei unterschiedlicher schriftlicher Aufklärung vorhanden sind, wird eine explorative Pilotstudie durchgeführt. Es sollen mindestens 20 Patienten pro Gruppe eingeschlossen werden, um sicherzustellen, dass belastbare Effektschätzer für die Fallzahlkalkulation einer Anschlussstudie gewonnen werden. Es wird angenommen, dass für etwa 15% keine Daten zum primären Endpunkt vorliegen werden (15% Lost-to-Follow-up). Daher sollen 23 Patienten pro Gruppe randomisiert werden, um sicherzustellen, dass 20 Patienten pro Gruppe mit vollständigen Daten analysiert werden können.

9.4 Statistischer Analyseplan

9.4. 1 Basischarakteristika

Die demografischen Daten und andere Basischarakteristika zur Beschreibung der Population werden deskriptiv aufbereitet. Für Angaben zu Intervall skalierten Variablen werden Mittelwert und Standardabweichung und für kategoriale Variablen absolute Häufigkeiten und Prozentwerte verwendet. Alle Angaben werden für die jeweilige Studiengruppe angegeben.

9.4. 2 Statistische Verfahren zur Endpunktauswertung

Zur Prüfung auf statistisch signifikante Unterschiede der Nebenwirkungen wird eine nicht prametrische Mean cumulative function (MCF) verwendet [32]. MCF erlaubt, die Analyse von wiederkehrenden Ereignissen über einen a-priori definierten Zeitraum. Die Ereignisse werden dabei kumuliert über den Zeitraum der Einnahme (+3 Tage) betrachtet. Um eine falsche Spezifikation des Modells zu vermeiden, werden die Mittelwerte und Ereignisratenfunktionen (Rate Function) direkt geschätzt, d.h. keine Verteilungsannahmen getroffen. Darüber hinaus hat diese Methode den Vorteil, dass die oftmals nicht zutreffende Annahme gleicher Zeitintervalle zwischen den Ereignissen nicht notwendig ist. Die Analyse wird adjustiert für die Minimisierungsvariablen (vgl. oben). Es werden sowohl die Interventionsgruppen jeweils mit der Kontrollgruppe verglichen, als auch die Interventionsgruppen untereinander (paarweise Vergleiche).

Zur Berücksichtigung der Multiplizität aufgrund des Vergleiches von mehr als zwei Gruppen werden für alle statistischen Vergleiche auf signifikante Unterschiede die Hypothesen a-priori geordnet. Eine Alphaadjustierung ist somit nicht notwendig [33].

Die Globalhypothese für den primären Endpunkt lautet somit:

H_0: Die Häufigkeiten der berichteten Nebenwirkungen ist in allen drei Gruppen gleich.

H_1: Es besteht ein Unterschied der Häufigkeiten der berichteten Nebenwirkungen bei mindestens einer der drei Behandlungsgruppen.

Wird diese abgelehnt, erfolgen die paarweisen Vergleiche. D.h. es werden die folgenden Hypothesen getestet.

1. $H_{0:}$ Es besteht kein Unterschied der Häufigkeiten der berichteten Nebenwirkungen zwischen der Gruppe, die eine neutrale Packungsbeilage erhält und der Kontrollgruppe (Standard-Packungsbeilage).
   $H_{1:}$ Es besteht ein Unterschied der Häufigkeiten der berichteten Nebenwirkungen zwischen der Gruppe, die eine neutrale Packungsbeilage erhält und der Kontrollgruppe (Standard-Packungsbeilage).
2. $H_{0:}$ Es besteht kein Unterschied der Häufigkeiten der berichteten Nebenwirkungen zwischen den beiden Interventionsgruppen (neutrale Packungsbeilage versus mündliche Aufklärung).
   $H_{1:}$ Es besteht ein Unterschied der Häufigkeiten der berichteten Nebenwirkungen zwischen den beiden Interventionsgruppen.

3. $H_{0:}$ Es besteht kein Unterschied der Häufigkeiten der berichteten Nebenwirkungen zwischen der Kontrollgruppe (Standard-Packungsbeilage) und der Gruppe, die eine mündliche Aufklärung erhält.
   $H_{1:}$ Es besteht ein Unterschied der Häufigkeiten der berichteten Nebenwirkungen zwischen der Kontrollgruppe und der Gruppe, die eine mündliche Aufklärung erhält.

Die deskriptive Beschreibung der Adhärenz erfolgt getrennt für die frühzeitige Beendigung der Einnahme und die korrekte Einnahme im Zeitraum zwischen Initiation und Beendigung der Einnahme (Implementations-Adhärenz) [34]. Es wird eine logistische Regression für den Vergleich der Adhärenz zwischen den Gruppen verwendet. Die logistische Regression wird für Baselineadhärenz, Alter, Bildungsstatus, Familienstand und geplante Behandlungsdauer der Schmerzmedikation stratifiziert.

Tests auf Unterschied bezüglich Zufriedenheit, Verständlichkeit und Angst werden mittels Analysis of Variance (ANOVA) adjustiert für Alter und Geschlecht durchgeführt.

Das Risiko von zusätzlichem Ressourcenverbrauch bzw. der Anteil an Patienten mit zusätzlichem Ressourcenverbrauch aufgrund von Nebenwirkungen wird als relatives Risiko (RR) beschrieben, wobei die Standard-Packungsbeilage (Kontrollgruppe) die Referenzgruppe ist. Falls ausreichend Beobachtung vorhanden sind, wird zudem ein Vergleich der Anzahl der Arztkontakte und ein Vergleich der eingenommen Arzneimittel mit dem Mann-Whitney-U-Test durchgeführt.

Alle statistischen Tests werden zu einem Signifikanzniveau von $\alpha=0.05$ durchgeführt und für die Punktschätzer der Effekt 95% Konfidenzintervalle angeben. Sämtliche statistische Test sind auf Grund des Pilotcharakters der Studie als rein explorativ anzusehen. Die gesamte Analyse wird mit SAS 9.4 durchgeführt.

9.4. 3 Subgruppenanalyse

Um zu untersuchen, ob ein Unterschied im primären Endpunkt bei Adhärente und Non-Adhärente Patienten besteht, wird eine Subgruppenanalyse durchgeführt. Hierfür wird ein Test auf Interaktion zwischen Adhärente bzw. Non-Adhärente Patienten und den Studiengruppen durchgeführt.

10. Datenmanagement

10.1 Patientenidentifikationsliste

Alle patientenbezogenen Daten werden in pseudonymisierter Form erfasst. Dazu wird ein Pseudonym verwendet, aus welchem allein nicht auf die Identität des Patienten geschlossen werden kann. Um eindeutige Patientenidentifikationsnummern zu erhalten, wird eine Kombination aus festgelegter fortlaufender Patientennummer gewählt. Die Patientenidentifikationsnummer wird im Rahmen der Randomisierung vom Studienzentrum zentral zugewiesen.

Jedes Prüfzentrum führt eine Patientenidentifikationsliste, in der die Patientenidentifikationsnummern mit den vollen Patientennamen der Teilnehmer, Patientenidentifikationsnummer und ggf. Geburtsdatum verbunden sind. Diese Liste dient der Möglichkeit der späteren Identifikation teilnehmender Personen. Diese wird absolut vertraulich behandelt und verbleibt ständig im Prüfzentrum.

10.2 Datenerhebung/Dokumentationsbögen

Zur Erreichung des Studienzieles ist es erforderlich, medizinische Daten einzelner Patienten zu erheben und zu verarbeiten. Die Datenerhebung erfolgt in dem, an der Studie beteiligten Zentrum. Die Datenerhebung erfolgt anhand von papierbasierten Dokumentationsbögen (paper based case report form [p-CRF]). Es wird für die Studien eine Studiendatenbank erstellt. In diese werden sämtliche Angaben aus dem CRF eingetragen. Zur Qualitätssicherung wird die Dateneingabe von einer zweiten Person überprüft. Die Datensätze werden regelmäßig abgeglichen und wenn nötig bereinigt.

10.3 Studienunterlagen und deren Aufbewahrung (Archivierung)

Die Originale aller essentiellen Studiendokumente werden beim Studienleiter für mindestens drei Jahre nach Erstellung des Abschlussberichtes aufbewahrt.
Alle Unterlagen werden in gesicherten Schränken aufbewahrt und vertraulich behandelt. Die Patientenidentifikationsliste wird getrennt von den Dokumentationsunterlagen aufbewahrt. Alle im Rahmen der Studie gewonnenen Daten werden auf einem gesicherten Server gespeichert. Es werden täglich Sicherungskopien erstellt.

10.4 Datenschutz

Im Rahmen der Studie ist es erforderlich, von den Studienteilnehmern personenbezogene Daten (z. B. vollständiger Name, Initialen des Vor- und Zunamens, Geburtsdatum, Adresse) und Daten zur Behandlung und zum Krankheitsverlauf (z. B. medizinische Befunde, Behandlungsarten, verordnete Medikamente) zu erheben und zu verarbeiten. Diese Daten werden in den Prüfzentren erhoben und in pseudonymisierter Form (d.h. ohne direkten Bezug zum Patientennamen) mit Hilfe einer

Patientenidentifikationsnummer elektronisch gespeichert. Die Bestimmungen des Bundes- und Landesdatenschutzgesetzes zum Datenschutz werden von allen Mitarbeitern beachtet.

Im Falle eines Widerrufs der Einwilligung zur Studie durch den Patienten einschließlich der weiteren Datenerhebung werden ab dem Widerrufszeitpunkt keine weiteren Daten erhoben. Die bisher erhobenen Daten werden innerhalb der Studie weiter verwendet und ausgewertet.

12. Ethische Belange, gesetzliche und administrative Regelungen

12.1 Deklaration von Helsinki und Gute klinische Praxis

Die gesamte Auswertung erfolgt gemäß ICH GCP (E9) und in Übereinstimmung mit der Deklaration von Helsinki in der aktuellen Version [1, 35].

Es sind für die Studie neben der Intervention und Patientenbefragung keine weiteren Maßnahmen und Untersuchungen am Menschen vorgesehen.

12.2 Ethik-Kommissionen

Ein positives Ethikvotum für die Studie liegt vor.

12.3 Nachträgliche Änderungen

Das Studienprotokoll ist einzuhalten. Jede vom Prüfer zu vertretende Abweichung von den vorgesehenen Untersuchungs- und Behandlungsmaßnahmen oder -zeitpunkten ist zu dokumentieren und zu begründen.

Änderungen oder Ergänzungen des Studienprotokolls können nur von der Studienleitung veranlasst und autorisiert werden. Über substantielle Änderungen des Studienprotokolls wird die federführende Ethikkommission informiert. Ggf. wird erneut die zustimmende Bewertung eingeholt. Bewertungspflichtige Änderungen (z.B. Änderung der neutralen Packungsbeilage) werden nicht vor der Entscheidung der Ethikkommission umgesetzt.

12.6 Registrierung

Die Studie soll in folgendem öffentlichen Register eingetragen werden: ClinicalTrials.gov“ und DRKS.
Für die Anmeldung im Register und die Pflege der Registerdaten ist Barbara Prediger zuständig.

12.7 Finanzierung

Die Studie wird durch die Deutsche Forschungsgemeinschafft finanziert (Projektnummer 338417115). Weiterhin besteht keine externe Finanzierung.

12.8 Abschlussbericht und Publikation

Alle Ergebnisse (z.B. Patientenfluss/Flow-Chart) werden entsprechend der Empfehlungen des CONSORT-Statements für nicht-medikamentöse-Studien und pragmatische Studien berichtet [36-38]. Die Beschreibung der Interventionen erfolgt in Übereinstimmung mit TIDieR (Better reporting of interventions: template for intervention description and replication (TIDieR) checklist and guide) berichtet [39].
Die Veröffentlichung der Studienergebnisse erfolgt unabhängig davon, wie die Ergebnisse ausfallen.

Es wird angestrebt die Ergebnisse in einem Open Access Journal zu veröffentlichen. Sämtliche Daten der Studie werden als elektronische Anhang der Publikation für weitere Forschungsvorhaben (z.B. IPD-Metaanalysen) zur Verfügung gestellt (pseudonymisierte Datensätze der Endauswertung etc.).

13. Literatur

1. INTERNATIONAL CONFERENCE ON HARMONISATION OF TECHNICAL REQUIREMENTS FOR REGISTRATION OF PHARMACEUTICALS FOR HUMAN USE: ICH HARMONISED TRIPARTITE GUIDELINE: GUIDELINE FOR GOOD CLINICAL PRACTICE E6(R1). In: *Current Step 4 version.* ICH; 1996.

2. Hauser W, Hansen E, Enck P: Nocebo phenomena in medicine: their relevance in everyday clinical practice. *Deutsches Arzteblatt international* 2012, 109(26):459-465.

3. Mitsikostas DD, Mantonakis L, Chalarakis N: Nocebo in clinical trials for depression: a meta-analysis. *Psychiatry research* 2014, 215(1):82-86.

4. Bingel U, Placebo Competence T: Avoiding nocebo effects to optimize treatment outcome. *Jama* 2014, 312(7):693-694.

5. World Health Organisation: ADHERENCE TO LONG-TERM THERAPIES-Evidence for action. 2003.

6. Dodd S, Schacht A, Kelin K, Duenas H, Reed VA, Williams LJ, Quirk FH, Malhi GS, Berk M: Nocebo effects in the treatment of major depression: results from an individual study participant-level meta-analysis of the placebo arm of duloxetine clinical trials. *The Journal of clinical psychiatry* 2015, 76(6):702-711.

7. Verdu F, Castello A: Non-compliance: a side effect of drug information leaflets. *Journal of medical ethics* 2004, 30(6):608-609.

8. Herber O, Gies V, Schwappach D, Thurmann P, Wilm S: Patient information leaflets: informing or frightening? A focus group study exploring patients' emotional reactions and subsequent behavior towards package leaflets of commonly prescribed medications in family practices. *BMC Family Practice* 2014, 15(1):163.

9. Howick J, Friedemann C, Tsakok M, Watson R, Tsakok T, Thomas J, Perera R, Fleming S, Heneghan C: Are treatments more effective than placebos? A systematic review and meta-analysis. *PloS one* 2013, 8(5):e62599.

10. Scott DJ, Stohler CS, Egnatuk CM, Wang H, Koeppe RA, Zubieta JK: Placebo and nocebo effects are defined by opposite opioid and dopaminergic responses. *Archives of general psychiatry* 2008, 65(2):220-231.

11. Amanzio M, Corazzini LL, Vase L, Benedetti F: A systematic review of adverse events in placebo groups of anti-migraine clinical trials. *Pain* 2009, 146(3):261-269.

12. Finegold JA, Manisty CH, Goldacre B, Barron AJ, Francis DP: What proportion of symptomatic side effects in patients taking statins are genuinely caused by the drug? Systematic review of randomized placebo-controlled trials to aid individual patient choice. *European journal of preventive cardiology* 2014, 21(4):464-474.

13. Max MB, Schafer SC, Culnane M, Dubner R, Gracely RH: Association of pain relief with drug side effects in postherpetic neuralgia: a single-dose study of clonidine, codeine, ibuprofen, and placebo. *Clinical pharmacology and therapeutics* 1988, 43(4):363-371.

14. Rojas-Mirquez JC, Rodriguez-Zuniga MJ, Bonilla-Escobar FJ, Garcia-Perdomo HA, Petkov M, Becerra L, Borsook D, Linnman C: Nocebo effect in randomized clinical trials of

antidepressants in children and adolescents: systematic review and meta-analysis. *Frontiers in behavioral neuroscience* 2014, 8:375.

15. Petersen GL, Finnerup NB, Colloca L, Amanzio M, Price DD, Jensen TS, Vase L: The magnitude of nocebo effects in pain: a meta-analysis. *Pain* 2014, 155(8):1426-1434.

16. Aslaksen PM, Zwarg ML, Eilertsen HI, Gorecka MM, Bjorkedal E: Opposite effects of the same drug: reversal of topical analgesia by nocebo information. *Pain* 2015, 156(1):39-46.

17. Mondaini N, Gontero P, Giubilei G, Lombardi G, Cai T, Gavazzi A, Bartoletti R: Finasteride 5 mg and sexual side effects: how many of these are related to a nocebo phenomenon? *The journal of sexual medicine* 2007, 4(6):1708-1712.

18. Varelmann D, Pancaro C, Cappiello EC, Camann WR: Nocebo-induced hyperalgesia during local anesthetic injection. *Anesthesia and analgesia* 2010, 110(3):868-870.

19. Myers MG, Cairns JA, Singer J: The consent form as a possible cause of side effects. *Clinical pharmacology and therapeutics* 1987, 42(3):250-253.

20. McGurk R, Fallowfield L, Winters Z: Information provision for patients by breast cancer teams about the side-effects of hormone treatments. *European Journal of Cancer* 2006, 42(12):1760-1767.

21. Schmitz J, Kamping S, Wiegratz J, Müller M, Stork J, Colloca L, Flor H, Klinger R: Impact of patient information leaflets on pain medication intake behavior: a pilot study. *PAIN Reports* 2017, 2(6):e620.

22. Weissenfeld J, Stock S, Lungen M, Gerber A: The nocebo effect: a reason for patients' non-adherence to generic substitution? *Die Pharmazie* 2010, 65(7):451-456.

23. Colloca L, Miller FG: The nocebo effect and its relevance for clinical practice. *Psychosomatic medicine* 2011, 73(7):598-603.

24. Scott NW, McPherson GC, Ramsay CR, Campbell MK: The method of minimization for allocation to clinical trials. a review. *Controlled clinical trials* 2002, 23(6):662-674.

25. Vambheim SM, Flaten MA: A systematic review of sex differences in the placebo and the nocebo effect. *Journal of pain research* 2017, 10:1831-1839.

26. Ancker JS, Senathirajah Y, Kukafka R, Starren JB: Design features of graphs in health risk communication: a systematic review. *Journal of the American Medical Informatics Association : JAMIA* 2006, 13(6):608-618.

27. Trevena LJ, Davey HM, Barratt A, Butow P, Caldwell P: A systematic review on communicating with patients about evidence. *Journal of evaluation in clinical practice* 2006, 12(1):13-23.

28. Buchter RB, Fechtelpeter D, Knelangen M, Ehrlich M, Waltering A: Words or numbers? Communicating risk of adverse effects in written consumer health information: a systematic review and meta-analysis. *BMC medical informatics and decision making* 2014, 14:76.

29. Bunge M, Muhlhauser I, Steckelberg A: What constitutes evidence-based patient information? Overview of discussed criteria. *Patient education and counseling* 2010, 78(3):316-328.

30. Schwappach DL, Mulders V, Simic D, Wilm S, Thurmann PA: Is less more? Patients' preferences for drug information leaflets. *Pharmacoepidemiology and drug safety* 2011, 20(9):987-995.

31. White IR, Royston P, Wood AM: Multiple imputation using chained equations: Issues and guidance for practice. *Statistics in medicine* 2011, 30(4):377-399.

32. Siddiqui O: Statistical methods to analyze adverse events data of randomized clinical trials. *Journal of biopharmaceutical statistics* 2009, 19(5):889-899.

33. Benjamini Y, Hochberg Y: Controlling the False Discovery Rate: A Practical and Powerful Approach to Multiple Testing. *Journal of the Royal Statistical Society Series B (Methodological)* 1995, 57(1):289-300.

34. Vrijens B, De Geest S, Hughes DA, Przemyslaw K, Demonceau J, Ruppar T, Dobbels F, Fargher E, Morrison V, Lewek P *et al*: A new taxonomy for describing and defining adherence to medications. *British journal of clinical pharmacology* 2012, 73(5):691-705.

35. World Medical Association Declaration of Helsinki: ethical principles for medical research involving human subjects. *Jama* 2013, 310(20):2191-2194.

36. Schulz KF, Altman DG, Moher D, Group C: CONSORT 2010 statement: updated guidelines for reporting parallel group randomised trials. *PLoS medicine* 2010, 7(3):e1000251.

37. Zwarenstein M, Treweek S, Gagnier JJ, Altman DG, Tunis S, Haynes B, Oxman AD, Moher D: Improving the reporting of pragmatic trials: an extension of the CONSORT statement. *BMJ* 2008, 337.

38. Boutron I, Altman DG, Moher D, Schulz KF, Ravaud P: CONSORT Statement for Randomized Trials of Nonpharmacologic Treatments: A 2017 Update and a CONSORT Extension for Nonpharmacologic Trial Abstracts. *Annals of internal medicine* 2017, 167(1):40-47.

39. Hoffmann TC, Glasziou PP, Boutron I, Milne R, Perera R, Moher D, Altman DG, Barbour V, Macdonald H, Johnston M *et al*: Better reporting of interventions: template for intervention description and replication (TIDieR) checklist and guide. *BMJ : British Medical Journal* 2014, 348.

14. Anlagen

A Case Report Form

CASE REPORT FORM

Version: 1.1 (31.01.2018)

Studie: Neutral formulierte Packungsbeilage oder ausschließlich mündliche Aufklärung gegenüber Packungsbeilage gemäß EU-Richtlinie: eine randomisiere kontrollierte Pilotstudie zur Analyse des Einflusses auf Nocebo-Effekt und Non-Adhärenz

[Protokoll V01-A01]

Generelle Anweisungen zum Ausfüllen des Prüffragebogens (CRF)

Allgemeines

- Es muss für jeden Patienten ein CRF ausgefüllt werden.
- Bitte füllen Sie den CRF in **Blockbuchstaben** aus unter Verwendung eines blauen oder schwarzen Kugelschreibers.
- Freitext und ergänzender Text sollte kurzgefasst werden.
- Alle Antworten sollten kurzgefasst werden ohne Verwendung von Wiederholungszeichen.
- Alle Fragen müssen beantwortet werden. Falls eine Antwort nicht bekannt ist bitte mit **„NB“** ausfüllen. Falls eine Frage nicht passt bitte mit **„NA“** ausfüllen.
- Falls Auswahlmöglichkeiten für eine Frage bestehen, wird die richtige Antwort angekreuzt **(X)**.

Daten und Zeiten

- Alle Datumsangaben müssen im Format TT.MM.JJJJ gemacht werden. (z.B. 01.01.2016).
- Falls kein genauer Zeitpunkt für ein Ereignis vorliegt, sollten die Zeitangaben wie folgt gemacht werden:

N	B	N	B	2	0	1	8
TT		MM		JJJJ			

- Falls nur eine Zeitspanne angeben werden kann, müssen Anfangs- bzw. Endpunkt der Zeitspanne gleichermaßen, wie oben beschrieben, angegeben werden.

Korrektur von Fehlern

- Einträge dürfen nicht überschrieben werden oder mit Korrekturflüssigkeiten überdeckt werden.
- Daten müssen durchgestrichen werden. Der korrigierte Eintrag sollte in direkter Nähe erfolgen.

BASELINE DATEN (ALLGEMEINE INFORMATIONEN UND PATIENTENCHARAKTERISTIKA)

TEILNEHMER INFORMATIONEN		
Probandenidentifikation	☐☐☐	
Einschluss-/Ausschlusskriterien<br>*Patient muss alle Kriterien erfüllen	Alle erfüllt ☐1.	Nicht alle erfüllt* ☐2.
Datum der Einwilligung	T T M M J J J J	
Telefonnummer	☐☐☐☐☐☐☐☐☐☐☐☐	
Geburtsdatum	T T M M J J J J	
Geschlecht	☐1 Frau<br>☐2 Mann	
Familienstand	☐1 Verheiratet/Partnerschafft<br>☐2 Ledig	
Schullabschluss	☐1 Noch in schulischer Ausbildung<br>☐2 Haupt-(Volks-)schulabschluss<br>☐3 Abschluss der polytechnischen Oberschule<br>☐4 Realschul- oder gleichwertiger Abschluss<br>☐5 Fachhochschul- oder Hochschulreife<br>☐6 Ohne allgemeinen Schulabschluss<br>☐7 Andere (bitte angeben):	
Berufliche Ausbildung	☐1 Ohne beruflichen Ausbildungsabschluss<br>☐2 Abschluss einer beruflichen Ausbildung von mindestens 1 Jahr<br>☐3 Hochschulabschluss	

BASELINE DATEN (MEDIZINISCH)

Erwerbstätigkeit	☐1 Ja<br>☐2 Nein
Einkommen	☐1 ≤2.000€<br>☐2 2.001€-2500€<br>☐3 2.501€-3.000€<br>☐4 3.001€-3.500€<br>☐5 3.501€-4.000€<br>☐6 4.001€-4.500€<br>☐7 5.501€-6.000€<br>☐8 6.001€-6.500€<br>☐9 ≥7.000€

TEILNEHMER INFORMATIONEN	
ICD	|_|_|_|_|_|_|
OPS	|_|_|_|_|_|_|
Angaben zu Ibuprofen	Dosierung pro Einnahme (Menge, Einheit):___________,____<br>Häufigkeit der Einnahme pro Tag: ________________________<br>Dauer der Einnahme (Tage): _____________________________

ENDPUNKT (MIT PACKUNGSBEILAGE)

**FINAL STUDY OUTCOME (bei Telefoninterview oder postalischer Befragung durch Studienassistenz auszufüllen)**	
**Datum Befragung/Rücksendung**	T T . M M . J J J J
Ausschließlich Ibuprofen eingenommen	☐1 Ja<br>☐2 Nein
**Angaben zur Packungsbeilage**	
Packungsbeilage gelesen	☐1 Ja<br>☐2 Nein
Verständlichkeit Packungsbeilage	☐
Wissen Wirkung	☐1 Ja<br>☐2 Nein
Wissen Nebenwirkung	☐1 Ja<br>☐2 Nein
Angst vor Nebenwirkungen	☐1 Ja<br>☐2 Nein
Stärke Angst	___________,_____ (eine Kommastelle)
Besonders positive	______________________<br>______________________
Besonders negativ	______________________<br>______________________

ENDPUNKT (MIT PACKUNGSBEILAGE)

**Angaben zu Medikamenteneinnahme**	
Dauer der Einnahme	☐1 ja<br>☐2 Nein, vergessen<br>☐3 Nein, Nebenwirkungen aufgetreten<br>☐4 Nein, Angst vor Nebenwirkungen<br>☐5 Nein, andere: ______________________________
Regelmäßige Einnahme	☐1 ja<br>☐2 Nein, vergessen<br>☐3 Nein, Nebenwirkungen aufgetreten<br>☐4 Nein, Angst vor Nebenwirkungen<br>☐5 Nein, andere: ______________________________
Zusätzliche Einnahme	☐1 Ja<br>☐2 Nein
**Angaben zu Nebenwirkungen**	
Nebenwirkungen	☐1 Ja<br>☐2 Nein
Magen-Darm-Beschwerden	☐1 Ja<br>☐2 Nein
Häufigkeit Magen-Darm-Beschwerden	☐1 1 mal<br>☐2 2 mal<br>☐3 3 mal<br>☐4 > 3mal
Stärke Magen-Darm-Beschwerden	____________,_____ (eine Kommastelle)

Störungen des zentralen Nervensystems	☐1 Ja<br>☐2 Nein
Häufigkeit Störungen des zentralen Nervensystems	☐1 1 mal<br>☐2 2 mal<br>☐3 3 mal<br>☐4 > 3mal
Stärke Störungen des zentralen Nervensystems	____________,____ (eine Kommastelle)
Andere Beschwerden	☐1 Ja<br>☐2 Nein<br>Beschreibung: ____
Ärztliche Behandlung	☐1 Ja<br>☐2 Nein
Medikamente wegen Nebenwirkungen	☐1 Ja<br>☐2 Nein<br>Beschreibung: ____

ENDPUNKT (OHNE PACKUNGSBEILAGE)

**FINAL STUDY OUTCOME (bei Telefoninterview oder postalischer Befragung durch Studienassistenz auszufüllen)**	
**Datum Befragung/Rücksendung**	T T M M J J J J
Ausschließlich Ibuprofen eingenommen	☐1 Ja<br>☐2 Nein
**Angaben zur Packungsbeilage**	
Andere Packungsbeilage gelesen	☐1 Ja<br>☐2 Nein
**Angaben zu Medikamenteneinnahme**	
Dauer der Einnahme	☐1 ja<br>☐2 Nein, vergessen<br>☐3 Nein, Nebenwirkungen aufgetreten<br>☐4 Nein, Angst vor Nebenwirkungen<br>☐5 Nein, andere: ____________________
Regelmäßige Einnahme	☐1 ja<br>☐2 Nein, vergessen<br>☐3 Nein, Nebenwirkungen aufgetreten<br>☐4 Nein, Angst vor Nebenwirkungen<br>☐5 Nein, andere: ____________________
Zusätzliche Einnahme	☐1 Ja<br>☐2 Nein
**Angaben zu Nebenwirkungen**	
Nebenwirkungen	☐1 Ja<br>☐2 Nein
Magen-Darm-Beschwerden	☐1 Ja<br>☐2 Nein
Häufigkeit Magen-Darm-Beschwerden	☐1 1 mal<br>☐2 2 mal<br>☐3 3 mal<br>☐4 > 3mal

Stärke Magen-Darm-Beschwerden	____________,____ (eine Kommastelle)
Störungen des zentralen Nervensystems	☐1 Ja<br>☐2 Nein
Häufigkeit Störungen des zentralen Nervensystems	☐1 1 mal<br>☐2 2 mal<br>☐3 3 mal<br>☐4 > 3mal
Stärke Störungen des zentralen Nervensystems	____________,____ (eine Kommastelle)
Andere Beschwerden	☐1 Ja<br>☐2 Nein<br>Beschreibung: ____
Ärztliche Behandlung	☐1 Ja<br>☐2 Nein
Medikamente wegen Nebenwirkungen	☐1 Ja<br>☐2 Nein<br>Beschreibung: ____

B Ethikvotum

Ethik-Kommission der
Universität Witten / Herdecke

Universität Witten/Herdecke - Ethik-Kommission · Alfred-Herrhausen-Str. 50 · D - 58448 Witten

Herrn
Dr. rer. medic. Tim Mathes
persönlich / vertraulich
Institut für Forschung in der Operativen Medizin (IFOM)
Haus 38
Ostmerheimer Str. 200
51109 Köln

Ethik-Kommission
Alfred-Herrhausen-Str. 50
D-58448 Witten

Sekretariat:
Frau Andrea Pleger
Mo-Fr 8.00-12.00 Uhr
Telefon 02302/926-740
Telefax 02302/926-739

e-mail: sekretariat-ethik@uni-wh.de
Internet: www.ethik-kommission-uwh.de

19.11.2015
Ga/eb

Antrag Nr. 170/2015 (bitte stets angeben):
Neutral formulierte Packungsbeilage oder ausschließlich mündliche Aufklärung gegenüber Packungsbeilage gemäß EU-Richtlinie: eine randomisierte kontrollierte Studie zur Analyse des Einflusses auf Nocebo-Effekt und Non-Adhärenz

Sehr geehrter Herr Dr. Mathes,

Ihre vorgenannte Studie lag der Ethik-Kommission der Universität Witten / Herdecke anlässlich ihrer Sitzung vom 11.11.2015 zur Beratung vor.

Grundsätzliche ethische oder berufsrechtliche Bedenken werden gegen die Studie sind nicht ersichtlich, die Kommission bittet jedoch um Beachtung folgender Hinweise:

Den Unterlagen war weder die original Packungsbeilage, noch die als Prüfgegenstand vorgesehene Version beigefügt. Beide sind nachzureichen.

Ferner möchte die Kommission eine Überprüfung der abgefragten Soziodemografischen Daten empfehlen. Im Begriff „Partnerschaft" haben sich orthographische Fehler eingeschlichen, der Familienstand „verwitwet" fehlt. Es werden diverse Schulabschlüsse abgefragt, nicht jedoch ein Hochschulabschluss.

Für die endgültige Beurteilung der Studie bittet die Kommission um Übersendung der vorstehend angesprochenen Unterlagen, ggf. nach deren Überarbeitung, in 1-facher Ausfertigung.

Mit freundlichen Grüßen

E. Barth

i. A.
RA Prof. Dr. med. P. W. Gaidzik
Geschäftsführendes Vorstandsmitglied

Anlage
Liste der Sitzungsteilnehmer

Ethik-Kommission der Universität Witten-Herdecke e. V.
Vorstand: Prof. Dr. med. Petra Thürmann (Vorsitzende), Prof. Dr. med. Hagen Tronnier, RA Prof. Dr. med. Peter W. Gaidzik
Sitz des Vereins: Witten, Amtsgericht Witten VR 779; Bank: Stadtsparkasse Witten (BLZ 452 500 35) Konto-Nr. 0050534
IBAN: DE 41 4525 0035 0000 0505 34 Swift-BIC: WELADED1WTN

Liste der Sitzungsteilnehmer an der Sitzung der Ethik-Kommission der Universität Witten / Herdecke vom 11.11.2015:

Herr RA Prof. Dr. med. Peter W. Gaidzik	Volljurist / Arzt (Vorsitz)
Frau PD Dr. med. Mozhgan Bizhang	Zahnmedizinerin
Herr Prof. Dr. rer. nat. Herbert Mayer	Mathematiker
Frau Prof. Dr. Martina Piefke	Patientenvertreterin
Herr Dr. med. Roland Raddatz	Anästhesist
Herr Prof. Dr. med. Dr. phil. nat. Achim Schmidtko	Klin. Pharmakologe / Apotheker
Herr Dr. theol. Rainer Schmitt	Theologe / Medizinethiker
