# Supplementary material for: Nocebo effects of a simplified package leaflet compared to unstandardised oral information and a standard package leaflet: a pilot randomised controlled trial
Source: Trials. 2019 Jul 26;20:458. doi: 10.1186/s13063-019-3565-3 (PMC6660653; doi:10.1186/s13063-019-3565-3)
Supplement: Supplementary file 2 — Changes to the protocol. (DOCX 12 kb) [file 13063_2019_3565_MOESM2_ESM.docx]

As per study protocol the primary outcome should be analyzed with a mean cumulative function. In this analysis the AEs are treated as count data (one patient can provide more than AE to the analysis) and similar to a survival analysis the time points between the events are incorporated in the analysis.

This analysis could not be performed because all patients experienced only 1 or 2 AEs or there was no event-free time between the AEs. For this reason we analyzed the number of patients who had any AE using logistic regression analysis.
