# Supplementary material for: Nocebo effects of a simplified package leaflet compared to unstandardised oral information and a standard package leaflet: a pilot randomised controlled trial
Source: Trials. 2019 Jul 26;20:458. doi: 10.1186/s13063-019-3565-3 (PMC6660653; doi:10.1186/s13063-019-3565-3)
Supplement: Supplementary file 3 — Simplified-PIL. (DOCX 48 kb) [file 13063_2019_3565_MOESM3_ESM.docx]

**Ibuprofen 600 mg film-coated tablets**

**Contents**

1. Mode of action and effectiveness
2. Application and duration of use
3. Side effects
4. Side effects that require stopping treatment or a visit to the doctor
5. Reasons to avoid taking ibuprofen and safety measures
6. Driving and operating machines
7. **Mode of action and effectiveness**

Ibuprofen helps to ease fever, acute pain and inflammation. It reduces the production of bodily substances that can cause these symptoms.

Studies show that ibuprofen can ease pain significantly in about 77 out of 100 people (pain is reduced by half or more after taking the drug). This number is equivalent to the green persons in the following graphic:

| 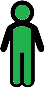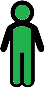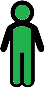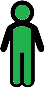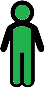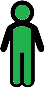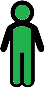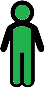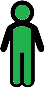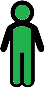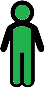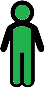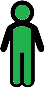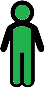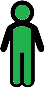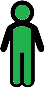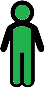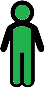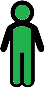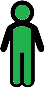  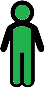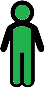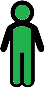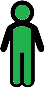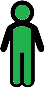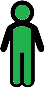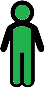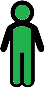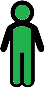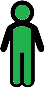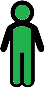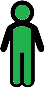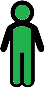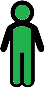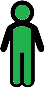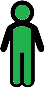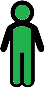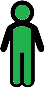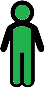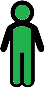  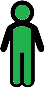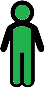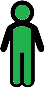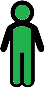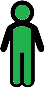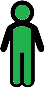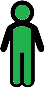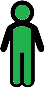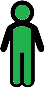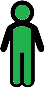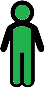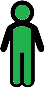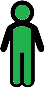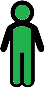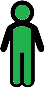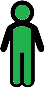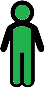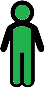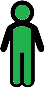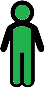  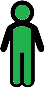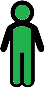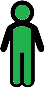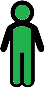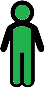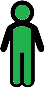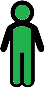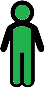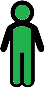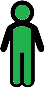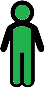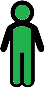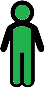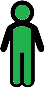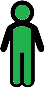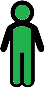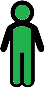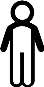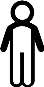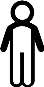  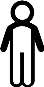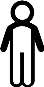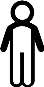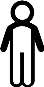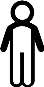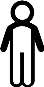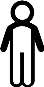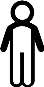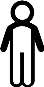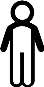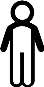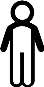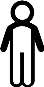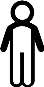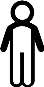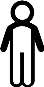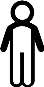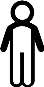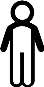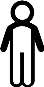 |
| --- |

1. **Application and duration of use**

Swallow the tablets whole (unchewed) with a glass of water. Avoid taking them on an empty stomach. Take ibuprofen as prescribed by your doctor.

If you have forgotten a dose, continue as prescribed (do not double dose to make up for a forgotten dose).

1. **Side effects**

Side effects do not necessarily occur for the entire treatment period and are not always much of a problem. Furthermore, most side effects disappear after finishing the treatment.

In studies, about 17 out of 100 people who took ibuprofen had side effects from the treatment.

In comparison, about 16 out of 100 people who took a placebo, reported side effects (a placebo is a fake pill that looks like the real one, but does not contain an active ingredient).

In other words: ibuprofen caused side effects in about 1 in 100 people. This is equivalent to the orange person in the following graphic. 99 out of 100 people did not have a side effect from taking ibuprofen (equaling the green persons in the graph):

|  |
| --- |

The most frequently reported side effects were:

- Stomach and bowel discomforts such as stomach ache, heartburn, nausea, being sick, diarrhea, flatulence or constipation
- Problems with the central nervous system such as headache, dizziness, sleeplessness, irritability or tiredness

**Gastrointestinal conditions**

|  |
| --- |

Ibuprofen caused stomach and bowel discomforts in about 1 in 100 people.
About 99 out of 100 people did not have stomach or bowel discomforts from ibuprofen.

**Side effects of the central nervous system**

Ibuprofen caused side effects of the central nervous system in less than 1 in 100 people.

Ibuprofen did not affect the central nervous system in more than 99 out of 100 people.

**Other side effects**

Some people reported other problems while taking ibuprofen. There is some evidence suggesting that ibuprofen may be connected to stomach or bowel bleeding, ulcers or perforations, and severe (in rare cases life-threatening) skin reactions. There are no good studies that provide information on the frequency of these side effects, however.

1. **Side effects that require stopping treatment or a visit to the doctor**

Stop taking ibuprofen, if you have severe stomach or bowel symptoms or ulcers.

These include bleeding in the stomach or bowel and problems with the liver or kidney function. Possible signs include low blood pressure (dizziness), a reduced breathing rate and purple skin color. See a doctor immediately, if such signs occur.

1. **Reasons to avoid taking ibuprofen and safety measures**

Do not take ibuprofen, if:

- you are allergic to one of the ingredients of Ibuprofen. These include: ibuprofen, croscarmellose sodium, hypromellose, macrogol 400, macrogol 600, magnesium stearate and maize starch.
- you have previously reacted to other painkillers that contain the same ingredients as ibuprofen such as acetylsalicylic acid (such as aspirin) with asthma attacks, nasal obstruction or skin reactions.
- you previously had blood disorders
- you previously had stomach or bowel bleeding or perforation due to ibuprofen or another painkiller with the same ingredients
- you currently have or previously had a stomach or bowel ulcer or bleeding
- you are suffering from bleeding on the brain (cerebral hemorrhage) or other bleeding
- you have a severe problem with the liver or kidney function
- you have heart failure
- you are in the last three months of pregnancy
- are under 15 years of age

**6. Driving and operating machines**

If you become tired or dizzy while taking ibuprofen, you should not drive or operate tools or machinery during this time.
