# Supplementary material for: Nocebo effects of a simplified package leaflet compared to unstandardised oral information and a standard package leaflet: a pilot randomised controlled trial
Source: Trials. 2019 Jul 26;20:458. doi: 10.1186/s13063-019-3565-3 (PMC6660653; doi:10.1186/s13063-019-3565-3)
Supplement: Supplementary file 4 — Standard-PIL. (PDF 253 kb) [file 13063_2019_3565_MOESM4_ESM.pdf]

GEBRAUCHSINFORMATION: Information für den Anwender

Ibuprofen STADA® 600 mg Filmtabletten

Wirkstoff: Ibuprofen

Lesen Sie die gesamte Packungsbeilage sorgfältig durch, bevor Sie mit der Einnahme dieses Arzneimittels beginnen.
- Heben Sie die Packungsbeilage auf. Vielleicht möchten Sie diese später nochmals lesen.
- Wenn Sie weitere Fragen haben, wenden Sie sich bitte an Ihren Arzt oder Apotheker.
- Dieses Arzneimittel wurde Ihnen persönlich verschrieben. Geben Sie es nicht an Dritte weiter. Es kann anderen Menschen schaden, auch wenn diese die gleichen Beschwerden haben wie Sie.
- Wenn eine der aufgeführten Nebenwirkungen Sie erheblich beeinträchtigt oder Sie Nebenwirkungen bemerken, die nicht in dieser Gebrauchsinformation angegeben sind, informieren Sie bitte Ihren Arzt oder Apotheker.

Diese Packungsbeilage beinhaltet:

1. Was ist Ibuprofen STADA® und wofür wird es angewendet?
2. Was müssen Sie vor der Einnahme von Ibuprofen STADA® beachten?
3. Wie ist Ibuprofen STADA® einzunehmen?
4. Welche Nebenwirkungen sind möglich?
5. Wie ist Ibuprofen STADA® aufzubewahren?
6. Weitere Informationen

1. Was ist Ibuprofen STADA® und wofür wird es angewendet?

Ibuprofen STADA® ist ein entzündungshemmendes und schmerzstillendes Arzneimittel (nichtsteroidales Antiphlogistikum/ Antirheumatikum)

Ibuprofen STADA® wird angewendet

zur symptomatischen Behandlung von Schmerz und Entzündung bei
- akuten Arthritiden (einschließlich Gichtanfall)
- chronischen Arthritiden, insbesondere bei rheumatoider Arthritis (chronische Polyarthritis)
- Spondylitis ankylosans (Morbus Bechterew) und anderen entzündlich-rheumatischen Wirbelsäulenerkrankungen
- Reizzuständen bei degenerativen Gelenk- und Wirbelsäulenerkrankungen (Arthrosen und Spondylarthrosen)
- entzündlichen weichteilrheumatischen Erkrankungen
- schmerzhaften Schwellungen und Entzündungen nach Verletzungen.

2. Was müssen Sie vor der Einnahme von Ibuprofen STADA® beachten?

Ibuprofen STADA® darf NICHT eingenommen werden
- wenn Sie überempfindlich (allergisch) gegen Ibuprofen oder einen der sonstigen Bestandteile von Ibuprofen STADA® sind
- wenn Sie in der Vergangenheit mit Asthmaanfällen, Nasenschleimhautschwellungen oder Hautreaktionen nach der Einnahme von Acetylsalicylsäure oder anderen nichtsteroidalen Entzündungshemmern reagiert haben
- bei ungeklärten Blutbildungsstörungen
- bei bestehenden oder in der Vergangenheit wiederholt aufgetretenen Magen- oder Zwölffingerdarm-Geschwüren (peptischen Ulzera) oder Blutungen (mindestens 2 unterschiedliche Episoden nachgewiesener Geschwüre oder Blutungen)
- bei Magen-Darm-Blutung oder -Durchbruch (Perforation) in der Vorgeschichte im Zusammenhang mit einer vorherigen Therapie mit nichtsteroidalen Antirheumatika/Antiphlogistika (NSAR)
- bei Hirnblutungen (zerebrovaskulären Blutungen) oder anderen aktiven Blutungen
- bei schweren Leber- oder Nierenfunktionsstörungen
- bei schwerer Herzmuskelschwäche (Herzinsuffizienz)
- im letzten Drittel der Schwangerschaft
- von Kindern und Jugendlichen unter 15 Jahren.

Im Folgenden wird beschrieben, wann Sie Ibuprofen STADA® nur unter bestimmten Bedingungen (d. h. in größeren Abständen oder in verminderter Dosis und unter ärztlicher Kontrolle) mit besonderer Vorsicht anwenden dürfen. Befragen Sie hierzu bitte Ihren Arzt.
Dies gilt auch, wenn diese Angaben bei Ihnen früher einmal zutrafen.

Besondere Vorsicht bei der Einnahme von Ibuprofen STADA® ist erforderlich
Sicherheit im Magen-Darm-Trakt
Eine gleichzeitige Anwendung von Ibuprofen STADA® mit anderen nichtsteroidalen Entzündungshemmern, einschließlich so genannten COX-2-Hemmern (Cyclooxygenase-2-Hemmern), sollte vermieden werden.

Nebenwirkungen können reduziert werden, indem die niedrigste wirksame Dosis über den kürzesten, für die Symptomkontrolle erforderlichen Zeitraum angewendet wird.

Ältere Patienten
Bei älteren Patienten treten häufiger Nebenwirkungen nach Anwendung von nichtsteroidalen Entzündungshemmern auf, insbesondere Blutungen und Durchbrüche im Magen- und Darmbereich, die unter Umständen lebensbedrohlich sein können. Daher ist bei älteren Patienten eine besonders sorgfältige ärztliche Überwachung erforderlich.

Blutungen des Magen-Darm-Trakts, Geschwüre und Durchbrüche (Perforationen)
Blutungen des Magen-Darm-Trakts, Geschwüre und Perforationen, auch mit tödlichem Ausgang, wurden unter allen NSAR berichtet. Sie traten mit oder ohne vorherige Warnsymptome bzw. schwerwiegende Ereignisse im Magen-Darm-Trakt in der Vorgeschichte zu jedem Zeitpunkt der Therapie auf.

Das Risiko für das Auftreten von Magen-Darm-Blutungen, Geschwüren und Durchbrüchen ist höher mit steigender NSAR-Dosis, in Patienten mit Geschwüren in der Vorgeschichte, insbesondere mit den Komplikationen Blutung oder Durchbruch (siehe unter Abschnitt 2: Ibuprofen STADA® darf NICHT eingenommen werden) und bei älteren Patienten. Diese Patienten sollten die Behandlung mit der niedrigsten verfügbaren Dosis beginnen.
Für diese Patienten sowie für Patienten, die eine begleitende Therapie mit niedrig dosierter Acetylsalicylsäure (ASS) oder anderen Arzneimitteln, die das Risiko für Magen-Darm-Erkrankungen erhöhen können, benötigen, sollte eine Kombinationstherapie mit Magenschleimhaut-schützenden Arzneimitteln (z. B. Misoprostol oder Protonenpumpenhemmer) in Betracht gezogen werden.

Wenn Sie eine Vorgeschichte von Nebenwirkungen am Magen-Darm- Trakt aufweisen, insbesondere in höherem Alter, sollten Sie jegliche ungewöhnliche Symptome im Bauchraum (vor allem Magen-Darm-Blutungen) insbesondere am Anfang der Therapie melden.

Vorsicht ist angeraten, wenn Sie gleichzeitig Arzneimittel erhalten, die das Risiko für Geschwüre oder Blutungen erhöhen können, wie z. B. zur Einnahme bestimmte Kortikosteroide, blutgerinnungshemmende Medikamente wie Warfarin, Selektive Serotoninwiederaufnahme- Hemmer, die unter anderem zur Behandlung von depressiven Verstimmungen eingesetzt werden, oder Thrombozytenaggregationshemmer (Blutgerinnungshemmer) wie Acetylsalicylsäure (siehe unter Abschnitt 2: Bei Einnahme von Ibuprofen STADA® mit anderen Arzneimitteln).

Wenn es bei Ihnen während der Behandlung mit Ibuprofen STADA® zu Magen-Darm-Blutungen oder Geschwüren kommt, ist die Behandlung abzusetzen.

NSAR sollten bei Patienten mit einer Magen-Darm-Erkrankung in der Vorgeschichte (Colitis ulcerosa, Morbus Crohn) mit Vorsicht angewendet werden, da sich ihr Zustand verschlechtern kann (siehe Abschnitt 4: Welche Nebenwirkungen sind möglich?).

Wirkungen am Herz-Kreislauf-System
Arzneimittel wie Ibuprofen STADA® sind möglicherweise mit einem geringfügig erhöhten Risiko für Herzanfälle (Herzinfarkt) oder Schlaganfälle verbunden. Jedwedes Risiko ist wahrscheinlicher mit hohen Dosen und länger dauernder Behandlung. Überschreiten Sie nicht die empfohlene Dosis oder Behandlungsdauer!

Wenn Sie Herzprobleme oder einen vorangegangenen Schlaganfall haben oder denken, dass Sie ein Risiko für diese Erkrankungen aufweisen könnten (z. B. wenn Sie hohen Blutdruck, Diabetes oder hohe Cholesterinwerte haben oder Raucher sind), sollten Sie Ihre Behandlung mit Ihrem Arzt oder Apotheker besprechen.

Hautreaktionen
Unter NSAR-Therapie wurde sehr selten über schwerwiegende Hautreaktionen mit Rötung und Blasenbildung, einige mit tödlichem Ausgang, berichtet (exfoliative Dermatitis, Stevens-Johnson-Syndrom und toxische epidermale Nekrolyse/Lyell-Syndrom; siehe Abschnitt 4: Welche Nebenwirkungen sind möglich?). Das höchste Risiko für derartige Reaktionen scheint zu Beginn der Therapie zu bestehen, da diese Reaktionen in der Mehrzahl der Fälle im ersten Behandlungsmonat auftraten. Beim ersten Anzeichen von Hautausschlägen, Schleimhautläsionen oder sonstigen Anzeichen einer Überempfindlichkeitsreaktion sollte Ibuprofen STADA® abgesetzt und umgehend der Arzt konsultiert werden.

Während einer Windpockeninfektion (Varizelleninfektion) sollte eine Anwendung von Ibuprofen STADA® vermieden werden.

Sonstige Hinweise
Ibuprofen STADA® sollte nur unter strenger Abwägung des Nutzen- Risiko-Verhältnisses angewendet werden
- bei bestimmten angeborenen Blutbildungsstörungen (z. B. akute intermittierende Porphyrie)
- bei bestimmten Autoimmunerkrankungen (systemischer Lupus erythematodes und Mischkollagenose).

Eine besonders sorgfältige ärztliche Überwachung ist erforderlich
- bei Magen-Darm-Beschwerden oder chronisch-entzündlichen Darmerkrankungen (Colitis ulcerosa, Morbus Crohn)
- bei Bluthochdruck oder Herzleistungsschwäche (Herzinsuffizienz)
- direkt nach größeren chirurgischen Eingriffen
- bei Allergien (z. B. Hautreaktionen auf andere Arzneimittel, Asthma, Heuschnupfen), chronischen Nasenschleimhautschwellungen (sog. Nasenpolypen) oder chronischen, die Atemwege verengenden Atemwegserkrankungen.
- bei eingeschränkter Nieren- oder Leberfunktion.

Schwere akute Überempfindlichkeitsreaktionen (zum Beispiel anaphylaktischer Schock) werden sehr selten beobachtet. Bei ersten Anzeichen einer Überempfindlichkeitsreaktion nach Einnahme von Ibuprofen STADA® muss die Therapie abgebrochen werden. Der Symptomatik entsprechende, medizinisch erforderliche Maßnahmen müssen durch fachkundige Personen eingeleitet werden.

Ibuprofen, der Wirkstoff von Ibuprofen STADA®, kann vorübergehend die Blutplättchenfunktion (Thrombozytenaggregation) hemmen.
Patienten mit Gerinnungsstörungen sollten daher sorgfältig überwacht werden.

Blutgerinnungshemmer (z. B. Acetylsalicylsäure, Warfarin, Ticlopidin), Arzneimittel gegen Bluthochdruck (ACE-Hemmer, z. B. Captopril, Betarezeptorblocker, Angiotensin-II-Antagonisten) sowie einige andere Arzneimittel können die Behandlung mit Ibuprofen beeinträchtigen oder durch eine solche selbst beeinträchtigt werden. Deshalb sollten Sie stets ärztlichen Rat einholen, bevor Sie Ibuprofen gleichzeitig mit anderen Arzneimitteln anwenden (siehe unter Abschnitt 2: Bei Einnahme von Ibuprofen STADA® mit anderen Arzneimitteln).

Wenn Sie gleichzeitig Arzneimittel zur Hemmung der Blutgerinnung oder zur Senkung des Blutzuckers einnehmen, sollten vorsichtshalber Kontrollen der Blutgerinnung bzw. der Blutzuckerwerte erfolgen.

Bei länger dauernder Gabe von Ibuprofen STADA® ist eine regelmäßige Kontrolle der Leberwerte, der Nierenfunktion sowie des Blutbildes erforderlich.

Bei Einnahme von Ibuprofen STADA® vor operativen Eingriffen ist der Arzt oder Zahnarzt zu befragen bzw. zu informieren.

Bei längerem Gebrauch von Schmerzmitteln können Kopfschmerzen auftreten, die nicht durch erhöhte Dosen des Arzneimittels behandelt werden dürfen. Fragen Sie Ihren Arzt um Rat, wenn Sie trotz der Einnahme von Ibuprofen STADA® häufig unter Kopfschmerzen leiden!

Ganz allgemein kann die gewohnheitsmäßige Einnahme von Schmerzmitteln, insbesondere bei Kombination mehrerer schmerzstillender Wirkstoffe, zur dauerhaften Nierenschädigung mit dem Risiko eines Nierenversagens (Analgetika-Nephropathie) führen.

Wie andere Arzneimittel, die die Prostaglandinsynthese hemmen, kann Ibuprofen STADA® es Ihnen erschweren, schwanger zu werden. Sie sollten Ihren Arzt informieren, wenn Sie planen schwanger zu werden oder wenn Sie Probleme haben, schwanger zu werden.

Kinder und Jugendliche
Kinder und Jugendliche unter 15 Jahren dürfen Ibuprofen STADA® nicht einnehmen, da der Wirkstoffgehalt zu hoch ist (siehe unter Abschnitt 2: Ibuprofen STADA® darf NICHT eingenommen werden). Für diese Altersgruppe stehen andere Ibuprofen-Zubereitungen mit geringerem Wirkstoffgehalt zur Verfügung.

Bei Einnahme von Ibuprofen STADA® mit anderen Arzneimitteln
Bitte informieren Sie Ihren Arzt oder Apotheker, wenn Sie andere Arzneimittel anwenden bzw. vor kurzem angewendet haben, auch wenn es sich um nicht verschreibungspflichtige Arzneimittel handelt.

Digoxin (Arzneimittel zur Stärkung der Herzkraft), Phenytoin (Arzneimittel zur Behandlung von Krampfanfällen) oder Lithium (Arzneimittel zur Behandlung geistig-seelischer Erkrankungen)
Die gleichzeitige Anwendung von Ibuprofen STADA® und Digoxin, Phenytoin oder Lithium kann die Konzentration dieser Arzneimittel im Blut erhöhen. Eine Kontrolle der Serum-Lithium-Spiegel ist nötig. Eine Kontrolle der Serum-Digoxin- und der Serum-Phenytoin-Spiegel wird empfohlen.

Entwässernde und blutdrucksenkende Arzneimittel (Diuretika und Antihypertensiva)
Ibuprofen STADA® kann die Wirkung von entwässernden und blutdrucksenkenden Arzneimitteln abschwächen.
Ibuprofen STADA® kann die Wirkung von ACE-Hemmern (Mittel zur Behandlung von Herzschwäche und Bluthochdruck) abschwächen. Bei gleichzeitiger Anwendung von ACE-Hemmern, Betarezeptorenblockern oder Angiotensin-II-Antagonisten (Mittel zur Behandlung von Herz- Kreislauf-Erkrankungen wie Bluthochdruck) kann weiterhin das Risiko für das Auftreten einer Nierenfunktionsstörung erhöht sein.

Kaliumsparende Entwässerungsmittel (bestimmte Diuretika) Die gleichzeitige Gabe von Ibuprofen STADA® und kaliumsparenden Entwässerungsmitteln kann zu einer Erhöhung des Kaliumspiegels im Blut führen.

Andere entzündungs- und schmerzhemmende Arzneimittel aus der Gruppe der nichtsteroidalen Antiphlogistika oder Glukokortikoide (Arzneimittel zur Behandlung von Entzündungen) Die gleichzeitige Verabreichung von Ibuprofen STADA® mit anderen entzündungs- und schmerzhemmenden Mitteln aus der Gruppe der nichtsteroidalen Antiphlogistika oder mit Glukokortikoiden erhöht das Risiko für Magen-Darm-Geschwüre oder Blutungen.

Thrombozytenaggregationshemmer (Blutgerinnungshemmer) wie Acetylsalicylsäure und bestimmte Antidepressiva (Selektive Serotoninwiederaufnahme-Hemmer/SSRI)
Thrombozytenaggregationshemmer wie Acetylsalicylsäure und bestimmte Antidepressiva (Selektive Serotoninwiederaufnahme- Hemmer/SSRI) können das Risiko für Magen-Darm-Blutungen erhöhen.

Niedrig dosierte Acetylsalicylsäure (zur Vorbeugung von Blutgerinnseln)
Untersuchungen deuten darauf hin, dass Ibuprofen bei gleichzeitiger Anwendung mit Acetylsalicylsäure die blutgerinnungshemmende Wirkung von niedrig dosierter Acetylsalicylsäure hemmen kann. Jedoch lassen sich keine sicheren Schlussfolgerungen bezüglich der regelmäßigen Anwendung von Ibuprofen treffen. Bei gelegentlicher Anwendung von Ibuprofen ist eine medizinisch bedeutsame Wechselwirkung nicht wahrscheinlich. Bitte sprechen Sie dennoch
vor der Einnahme von Ibuprofen STADA® mit Ihrem Arzt, wenn Sie regelmäßig niedrig dosierte Acetylsalicylsäure-haltige Arzneimittel zur Blutgerinnungshemmung einnehmen.

Blutgerinnungshemmende Arzneimittel wie Warfarin
NSAR können möglicherweise die Wirkung von blutgerinnungshemmenden Arzneimitteln wie Warfarin verstärken. Bei gleichzeitiger Behandlung wird eine Kontrolle des Gerinnungsstatus empfohlen.

Methotrexat (Arzneimittel zur Behandlung von Krebserkrankungen bzw. von bestimmten rheumatischen Erkrankungen)
Die Einnahme von Ibuprofen STADA® innerhalb von 24 Stunden vor oder nach Gabe von Methotrexat kann zu einer erhöhten Konzentration von Methotrexat und einer Zunahme seiner unerwünschten Wirkungen führen.

Ciclosporin (Arzneimittel, das zur Verhinderung von Transplantatabstoßungen, aber auch in der Rheumabehandlung eingesetzt wird)
Das Risiko einer nierenschädigenden Wirkung durch Ciclosporin wird durch die gleichzeitige Gabe bestimmter nichtsteroidaler Antiphlogistika erhöht. Dieser Effekt kann auch für eine Kombination von Ciclosporin mit Ibuprofen nicht ausgeschlossen werden.

Probenecid oder Sulfinpyrazon (Arzneimittel zur Behandlung von Gicht)
Arzneimittel, die Probenecid oder Sulfinpyrazon enthalten, können die Ausscheidung von Ibuprofen verzögern. Dadurch kann es zu einer Anreicherung von Ibuprofen im Körper mit Verstärkung seiner unerwünschten Wirkungen kommen.

Sulfonylharnstoffe (Arzneimittel zur Senkung des Blutzuckers) Klinische Untersuchungen haben Wechselwirkungen zwischen nichtsteroidalen Antiphlogistika und Sulfonylharnstoffen gezeigt. Obwohl Wechselwirkungen zwischen Ibuprofen und Sulfonylharnstoffen bisher nicht beschrieben sind, wird vorsichtshalber bei gleichzeitiger Einnahme eine Kontrolle der Blutzuckerwerte empfohlen.

Tacrolimus (Arzneimittel u. a. zur Verhinderung von Transplantatabstoßungen)
Das Risiko einer Nierenschädigung ist erhöht, wenn beide Arzneimittel gleichzeitig verabreicht werden.

Zidovudin (Arzneimittel zur Behandlung von HIV-Infektionen)
Es gibt Hinweise auf ein erhöhtes Risiko für Einblutungen in Gelenke (Hämarthrosen) und Hämatome bei HIV-positiven Hämophilie-Patienten („Blutern“), die gleichzeitig Zidovudin und Ibuprofen einnehmen.

Bei Einnahme von Ibuprofen STADA® zusammen mit Nahrungsmitteln und Getränken
Während der Anwendung von Ibuprofen STADA® sollten Sie möglichst keinen Alkohol trinken.

Schwangerschaft und Stillzeit
Fragen Sie vor der Anwendung von allen Arzneimitteln Ihren Arzt oder Apotheker um Rat.

Schwangerschaft
Wird während der Anwendung von Ibuprofen STADA® eine Schwangerschaft festgestellt, so ist der Arzt zu benachrichtigen. Sie dürfen Ibuprofen STADA® im ersten und zweiten Schwangerschaftsdrittel nur nach Rücksprache mit Ihrem Arzt anwenden.

Im letzten Drittel der Schwangerschaft darf Ibuprofen STADA® wegen eines erhöhten Risikos von Komplikationen für Mutter und Kind nicht angewendet werden.

Stillzeit
Der Wirkstoff Ibuprofen und seine Abbauprodukte gehen nur in geringen Mengen in die Muttermilch über. Da nachteilige Folgen für den Säugling bisher nicht bekannt geworden sind, wird bei kurzfristiger Anwendung eine Unterbrechung des Stillens in der Regel nicht erforderlich sein. Wird eine längere Anwendung bzw. Einnahme höherer Dosen verordnet, sollte jedoch ein frühzeitiges Abstillen erwogen werden.

Verkehrstüchtigkeit und das Bedienen von Maschinen
Da bei der Anwendung von Ibuprofen STADA® in höherer Dosierung zentralnervöse Nebenwirkungen wie Müdigkeit und Schwindel auftreten können, kann im Einzelfall das Reaktionsvermögen verändert und die Fähigkeit zur aktiven Teilnahme am Straßenverkehr und zum Bedienen von Maschinen beeinträchtigt werden. Dies gilt in verstärktem Maße im Zusammenwirken mit Alkohol. Sie können dann auf unerwartete und plötzliche Ereignisse nicht mehr schnell und gezielt genug reagieren. Fahren Sie in diesem Fall nicht Auto oder andere Fahrzeuge! Bedienen Sie keine Werkzeuge oder Maschinen! Arbeiten Sie nicht ohne sicheren Halt!

3. Wie ist Ibuprofen STADA® einzunehmen?

Nehmen Sie Ibuprofen STADA® immer genau nach der Anweisung des Arztes ein. Bitte fragen Sie bei Ihrem Arzt oder Apotheker nach, wenn Sie sich nicht ganz sicher sind.

Falls vom Arzt nicht anders verordnet, ist die übliche Dosis
<u>zur Therapie rheumatischer Erkrankungen:</u>
Ibuprofen wird in Abhängigkeit vom Alter bzw. Körpergewicht dosiert. Der empfohlene Dosisbereich für Erwachsene und Jugendliche ab
15 Jahren liegt zwischen 1200 und 2400 mg Ibuprofen pro Tag. Die maximale Einzeldosis für Erwachsene sollte höchstens 800 mg Ibuprofen betragen.

Alter	Einzeldosis	Tagesgesamtdosis
Jugendliche ab 15 Jahren und Erwachsene	½ bis 1 Tablette (entsprechend 300–600 mg Ibuprofen)	2 bis 4 Tabletten (entsprechend 1200–2400 mg Ibuprofen)

Art und Dauer der Anwendung
Nehmen Sie Ibuprofen STADA® unzerkaut mit reichlich Flüssigkeit und nicht auf nüchternen Magen ein. Wenn Sie einen empfindlichen Magen haben, empfiehlt es sich, Ibuprofen STADA® während der Mahlzeiten einzunehmen.

Bei rheumatischen Erkrankungen kann die Einnahme von Ibuprofen STADA® über einen längeren Zeitraum erforderlich sein. Über die Dauer der Anwendung entscheidet der behandelnde Arzt.

Bitte sprechen Sie mit Ihrem Arzt oder Apotheker, wenn Sie den Eindruck haben, dass die Wirkung von Ibuprofen STADA® zu stark oder zu schwach ist.

Wenn Sie eine größere Menge Ibuprofen STADA® eingenommen haben, als Sie sollten
Nehmen Sie Ibuprofen STADA® nach den Anweisungen des Arztes bzw. nach der in der Packungsbeilage angegebenen Dosierungsanleitung ein. Wenn Sie das Gefühl haben, keine ausreichende Schmerzlinderung zu spüren, dann erhöhen Sie nicht selbständig die Dosierung, sondern fragen Sie Ihren Arzt.

Folgende Symptome können bei einer Überdosierung auftreten:
- zentralnervöse Störungen wie Kopfschmerzen, Schwindel, Benommenheit und Bewusstlosigkeit (bei Kindern auch myoklonische Krämpfe)
- Bauchschmerzen
- Übelkeit
- Erbrechen
- Blutungen im Magen-Darm-Trakt
- Funktionsstörungen von Leber und Nieren
- Blutdruckabfall
- verminderte Atmung (Atemdepression)
- blaurote Färbung von Haut und Schleimhäuten (Zyanose).

Es gibt kein spezifisches Gegenmittel (Antidot).

Bei Verdacht auf eine Überdosierung mit Ibuprofen STADA® benachrichtigen Sie bitte Ihren Arzt. Dieser kann entsprechend der Schwere einer Vergiftung über die gegebenenfalls erforderlichen Maßnahmen entscheiden.

Wenn Sie die Einnahme von Ibuprofen STADA® vergessen haben
Falls Sie die Einnahme einmal vergessen haben, nehmen Sie bei der nächsten Gabe nicht mehr als die übliche empfohlene Menge ein.

Wenn Sie weitere Fragen zur Anwendung des Arzneimittels haben, fragen Sie Ihren Arzt oder Apotheker.

4. Welche Nebenwirkungen sind möglich?

Wie alle Arzneimittel kann Ibuprofen STADA® Nebenwirkungen haben, die aber nicht bei jedem auftreten müssen. Sollten Sie die folgenden Nebenwirkungen bei sich beobachten, besprechen Sie das bitte mit Ihrem Arzt, der dann festlegt, wie weiter zu verfahren ist.

Bei der Bewertung von Nebenwirkungen werden folgende Häufigkeitsangaben zugrunde gelegt:

Sehr häufig:	mehr als 1 Behandelter von 10
Häufig:	1 bis 10 Behandelte von 100
Gelegentlich:	1 bis 10 Behandelte von 1.000
Selten:	1 bis 10 Behandelte von 10 000
Sehr selten:	weniger als 1 Behandelter von 10 000
Nicht bekannt:	Häufigkeit auf Grundlage der verfügbaren Daten nicht abschätzbar

Mögliche Nebenwirkungen

Bei den folgenden unerwünschten Arzneimittelwirkungen muss berücksichtigt werden, dass sie überwiegend dosisabhängig und von Patient zu Patient unterschiedlich sind.

Die am häufigsten beobachteten Nebenwirkungen betreffen den Verdauungstrakt. Magen- oder Zwölffingerdarm-Geschwüre (peptische Ulzera), Perforationen (Durchbrüche) oder Blutungen, manchmal tödlich, können auftreten, insbesondere bei älteren Patienten (siehe unter Abschnitt 2: Besondere Vorsicht bei der Einnahme von Ibuprofen STADA® ist erforderlich). Übelkeit, Erbrechen, Durchfall, Blähungen, Verstopfung, Verdauungsbeschwerden, Bauchschmerzen, Teerstuhl, Bluterbrechen, Mundschleimhautentzündung (ulzerative Stomatitis), Verschlimmerung von Colitis und Morbus Crohn (siehe unter Abschnitt 2: Besondere Vorsicht bei der Einnahme von Ibuprofen STADA® ist erforderlich) sind nach Anwendung berichtet worden. Weniger häufig wurde Magenschleimhautentzündung beobachtet. Insbesondere das Risiko für das Auftreten von Magen-Darm-Blutungen ist abhängig vom Dosisbereich und der Anwendungsdauer.

Ödeme, Bluthochdruck und Herzinsuffizienz wurden im Zusammenhang mit NSAR-Behandlung berichtet.

Arzneimittel wie Ibuprofen STADA® sind möglicherweise mit einem geringfügig erhöhten Risiko für Herzanfälle (Herzinfarkt) oder Schlaganfälle verbunden.

Herzerkrankungen
Sehr selten: Herzklopfen (Palpitationen), Flüssigkeitsansammlung im Gewebe (Ödeme), Herzmuskelschwäche (Herzinsuffizienz), Herzinfarkt.

Erkrankungen des Blutes und des Lymphsystems
Sehr selten: Störungen der Blutbildung (Anämie, Leukopenie, Thrombozytopenie, Panzytopenie, Agranulozytose).
Erste Anzeichen können sein: Fieber, Halsschmerzen, oberflächliche Wunden im Mund, grippeartige Beschwerden, starke Abgeschlagenheit, Nasenbluten und Hautblutungen.
In diesen Fällen ist das Arzneimittel sofort abzusetzen und der Arzt aufzusuchen. Jegliche Selbstbehandlung mit schmerz- oder fiebersenkenden Arzneimitteln sollte unterbleiben.

Bei Langzeittherapie sollte das Blutbild regelmäßig kontrolliert werden.

Erkrankungen des Nervensystems
Häufig: Zentralnervöse Störungen wie Kopfschmerzen, Schwindel, Schlaflosigkeit, Erregung, Reizbarkeit oder Müdigkeit.

Augenerkrankungen
Gelegentlich: Sehstörungen.

Erkrankungen des Ohrs und des Labyrinths
Sehr selten: Ohrgeräusche (Tinnitus), Hörstörungen.

Erkrankungen des Magen-Darm-Trakts
Sehr häufig: Magen-Darm-Beschwerden wie Sodbrennen, Bauchschmerzen, Übelkeit, Erbrechen, Blähungen, Durchfall, Verstopfung und geringfügige Magen-Darm-Blutverluste, die in Ausnahmefällen eine Blutarmut (Anämie) verursachen können.
Häufig: Magen-/Zwölffingerdarm-Geschwüre (peptische Ulzera), unter Umständen mit Blutung und Durchbruch. Mundschleimhautentzündung mit Geschwürbildung (ulzerative Stomatitis), Verstärkung einer Colitis ulcerosa oder eines Morbus Crohn.
Gelegentlich: Magenschleimhautentzündung (Gastritis).
Sehr selten: Entzündung der Speiseröhre (Ösophagitis) und der Bauchspeicheldrüse (Pankreatitis), Ausbildung von membranartigen Verengungen in Dünn- und Dickdarm (intestinale, diaphragmaartige Strukturen).

Sollten stärkere Schmerzen im Oberbauch, Bluterbrechen, Blut im Stuhl und/oder eine Schwarzfärbung des Stuhls auftreten, so müssen Sie Ibuprofen STADA® absetzen und sofort den Arzt informieren.

Erkrankungen der Nieren und Harnwege
Gelegentlich: Vermehrte Wassereinlagerung im Gewebe mit Ausbildung von Ödemen, insbesondere bei Patienten mit Bluthochdruck oder eingeschränkter Nierenfunktion; nephrotisches Syndrom (Wasseransammlung im Körper [Ödeme] und starke Eiweißausscheidung im Harn); entzündliche Nierenerkrankung (interstitielle Nephritis), die mit einer akuten Nierenfunktionsstörung einhergehen kann.
Sehr selten können Nierengewebsschädigungen (Papillennekrosen) und erhöhte Harnsäurekonzentrationen im Blut auftreten.

Verminderung der Harnausscheidung, Ansammlung von Wasser im Körper (Ödeme) sowie allgemeines Unwohlsein können Ausdruck einer Nierenerkrankung bis hin zum Nierenversagen sein.
Sollten die genannten Symptome auftreten oder sich verschlimmern, so müssen Sie Ibuprofen STADA® absetzen und sofort Kontakt mit Ihrem Arzt aufnehmen.

Erkrankungen der Haut und des Unterhautzellgewebes
Sehr selten: Schwere Hautreaktionen wie Hautausschlag mit Rötung und Blasenbildung (z. B. Stevens-Johnson-Syndrom, toxische epidermale Nekrolyse/Lyell-Syndrom), Haarausfall (Alopezie).

In Ausnahmefällen kann es zu einem Auftreten von schweren Hautinfektionen und Weichteilkomplikationen während einer Windpockenerkrankung (Varizelleninfektion) kommen (siehe auch Infektionen und parasitäre Erkrankungen).

Infektionen und parasitäre Erkrankungen
Sehr selten ist im zeitlichen Zusammenhang mit der Anwendung bestimmter entzündungshemmender Arzneimittel (nichtsteroidaler Antiphlogistika; zu diesen gehört auch Ibuprofen STADA®) eine Verschlechterung infektionsbedingter Entzündungen (z. B. Entwicklung einer nekrotisierenden Fasziitis) beschrieben worden.
Wenn während der Anwendung von Ibuprofen STADA® Zeichen einer Infektion (z. B. Rötung, Schwellung, Überwärmung, Schmerz, Fieber) neu auftreten oder sich verschlimmern, sollte daher unverzüglich der Arzt zu Rate gezogen werden.
Sehr selten wurde unter der Anwendung von Ibuprofen die Symptomatik einer nicht auf einer Infektion beruhenden Hirnhautentzündung (aseptischen Meningitis) wie starke Kopfschmerzen, Übelkeit, Erbrechen, Fieber, Nackensteifigkeit oder Bewusstseinstrübung beobachtet. Ein erhöhtes Risiko scheint für Patienten zu bestehen, die bereits an bestimmten Autoimmunerkrankungen (systemischer Lupus erythematodes, Mischkollagenosen) leiden.

Gefäßerkrankungen
Sehr selten: Bluthochdruck (arterielle Hypertonie).

Erkrankungen des Immunsystems
Gelegentlich: Überempfindlichkeitsreaktionen mit Hautausschlägen und Hautjucken sowie Asthmaanfällen (ggf. mit Blutdruckabfall).
In diesem Fall ist umgehend der Arzt zu informieren und Ibuprofen STADA® darf nicht mehr eingenommen werden.

Sehr selten: Schwere allgemeine Überempfindlichkeitsreaktionen. Sie können sich äußern als: Schwellungen von Gesicht, Zunge und innerem Kehlkopf mit Einengung der Luftwege, Luftnot, Herzjagen, Blutdruckabfall bis hin zum bedrohlichen Schock.
Beim Auftreten einer dieser Erscheinungen, die schon bei Erstanwendung vorkommen können, ist sofortige ärztliche Hilfe erforderlich. Der Patient ist anzuweisen, in diesem Fall umgehend den Arzt zu informieren und Ibuprofen STADA® nicht mehr einzunehmen.

Leber- und Gallenerkrankungen
Sehr selten: Leberfunktionsstörungen, Leberschäden, insbesondere bei der Langzeittherapie, Leberversagen, akute Leberentzündung (Hepatitis).
Bei länger dauernder Gabe sollten die Leberwerte regelmäßig kontrolliert werden.

Psychiatrische Erkrankungen
Sehr selten: Psychotische Reaktionen, Depression.

Meldung von Nebenwirkungen
Wenn Sie Nebenwirkungen bemerken, wenden Sie sich an Ihren Arzt oder Apotheker. Dies gilt auch für Nebenwirkungen, die nicht in dieser Packungsbeilage angegeben sind. Sie können Nebenwirkungen auch direkt anzeigen:
Bundesinstitut für Arzneimittel und Medizinprodukte Abt. Pharmakovigilanz
Kurt-Georg-Kiesinger-Allee 3 D-53175 Bonn
Website: www.bfarm.de

Indem Sie Nebenwirkungen melden, können Sie dazu beitragen, dass mehr Informationen über die Sicherheit dieses Arzneimittels zur Verfügung gestellt werden.

5. Wie ist Ibuprofen STADA® aufzubewahren?

Arzneimittel für Kinder unzugänglich aufbewahren.

Sie dürfen das Arzneimittel nach dem auf der Faltschachtel und der Durchdrückpackung angegebenen Verfallsdatum nicht mehr anwenden.

Für dieses Arzneimittel sind keine besonderen Lagerungsbedingungen erforderlich.

Arzneimittel sollten nicht im Abwasser oder Haushaltsabfall entsorgt werden. Fragen Sie Ihren Apotheker wie das Arzneimittel zu entsorgen ist, wenn Sie es nicht mehr benötigen. Diese Maßnahme hilft die Umwelt zu schützen.

6. Weitere Informationen

Was Ibuprofen STADA® 600 mg enthält
Der Wirkstoff ist: Ibuprofen.

1 Filmtablette enthält 600 mg Ibuprofen.

Die sonstigen Bestandteile sind
Carboxymethylstärke-Natrium (Typ A) (Ph.Eur.), Hypromellose, Macrogol 400, Macrogol 6000, Magnesiumstearat (Ph.Eur.), Maisstärke.

Wie Ibuprofen STADA® aussieht und Inhalt der Packung Weiße, oblonge, bikonvexe Filmtablette mit beidseitiger Bruchrille. Die Tablette kann in gleiche Hälften geteilt werden.
Ibuprofen STADA® 600 mg ist in Packungen mit 20, 50 und 100 Filmtabletten erhältlich.

Pharmazeutischer Unternehmer
STADApharm GmbH Stadastraße 2–18
61118 Bad Vilbel
Telefon: 06101 603-0
Telefax: 06101 603-259 Internet: www.stada.de

Hersteller
STADA Arzneimittel AG Stadastraße 2–18
61118 Bad Vilbel

Diese Gebrauchsinformation wurde zuletzt überarbeitet im Mai 2014.

9263260
1408

STADA
